# Wnt Secretion Is Regulated by the Tetraspan Protein HIC-1 through Its Interaction with Neurabin/NAB-1

**DOI:** 10.1016/j.celrep.2018.10.053

**Published:** 2018-11-13

**Authors:** Vina Tikiyani, Lei Li, Pallavi Sharma, Haowen Liu, Zhitao Hu, Kavita Babu

**Affiliations:** 1 Indian Institute of Science Education and Research (IISER) Mohali, Knowledge City, Sector 81, SAS Nagar, Manauli PO 140306, Punjab, India; 2 Queensland Brain Institute, Clem Jones Centre for Ageing Dementia Research (CJCADR), University of Queensland, Upland Road 79, St. Lucia, QLD 4072, Australia

## Abstract

The aberrant regulation of Wnt secretion is implicated in various neurological diseases. However, the mechanisms of Wnt release are still largely unknown. Here we describe the role of a *C. elegans* tetraspan protein, HIC-1, in maintaining normal Wnt release. We show that HIC-1 is expressed in cholinergic synapses and that mutants in *hic-1* show increased levels of the acetylcholine receptor AChR/ACR-16. Our results suggest that HIC-1 maintains normal AChR/ACR-16 levels by regulating normal Wnt release from presynaptic neurons, as *hic-1* mutants show an increase in secreted Wnt from cholinergic neurons. We further show that HIC-1 affects Wnt secretion by modulating the actin cytoskeleton through its interaction with the actin-binding protein NAB-1. In summary, we describe a protein, HIC-1, that functions as a neuromodulator by affecting postsynaptic AChR/ACR-16 levels by regulating presynaptic Wnt release from cholinergic motor neurons.

## Introduction

Cell adhesion molecules (CAMs) are involved in functioning of neurons and synapses (reviewed in Abbas, 2003; Tallafuss et al., 2010; Yamagata et al., 2003). Claudins are one such class of tetraspan CAMs that are important structural and functional components of tight junctions and are known to maintain epithelial and endothelial tissue integrity and barrier functions (reviewed in Tsukita and Furuse, 2000).

The claudin superfamily of proteins is conserved structurally but is highly divergent at the sequence level (reviewed in Hua et al., 2003; Krause et al., 2008b). A growing body of evidence suggests functions for claudins in the brain because they are essential components of the blood-brain barrier, and their deregulation is associated with various brain disorders (reviewed in Gonçalves et al., 2013). Most claudins possess a PDZ binding motif at their C-terminal tail by which they interact with PDZ domain-containing scaffolding proteins that in turn act as adaptors that link claudins to the actin cytoskeleton in epithelial cells (reviewed in Günzel and Yu, 2013). How claudins and other tetraspan proteins function at synapses is largely unknown. We show that a claudin-like molecule, HIC-1, functions intracellularly like a claudin at the *C. elegans* neuromuscular junction, where it interacts with the actin cytoskeleton through the PDZ domain-containing, actin-binding protein Neurabin/NAB-1.

Wnt secretory proteins are conserved across the animal kingdom. Wnt signaling regulates various aspects of animal development, including development of the CNS. Aberrant regulation of this pathway is the cause of various diseases, such as cancers, fibrosis, and neurodegeneration (reviewed in Kahn, 2014). Much previous work has focused primarily on identifying molecules and their mechanism of action in the Wnt signaling pathways in different tissues (reviewed in Hussaini et al., 2014; Maguschak and Ressler, 2012; Veltri et al., 2017). However, studies of the secretion of Wnt ligands themselves have lagged behind. These studies have recently gained momentum because abnormal Wnt release is seen in an increasing number of diseases (reviewed in Herr et al., 2012). The Wnt signaling pathway that regulates AChR/ACR-16 delivery onto the body-wall muscles has been well characterized in *C. elegans* (Babu et al., 2011; Francis et al., 2005; Jensen et al., 2012; Pandey et al., 2017), but the mechanism by which Wnt secretion is regulated from *C. elegans* motor neurons in order to affect postsynaptic AChR/ACR-16 levels is still unknown. Our data show that HIC-1 is required to modulate Wnt secretion.

Although little work has been done detailing the mechanisms of Wnt release at synaptic sites, Wnt exosomes are thought to be in the proximity of F-actin at the *Drosophila* NMJ (reviewed in Koles and Budnik, 2012a, 2012b). However, the role of the actin cytoskeleton in mediating Wnt release has not been sufficiently investigated. In this study, we show that HIC-1 regulates Wnt release by modulating the presynaptic actin cytoskeleton, through its interaction with the actin-binding protein Neurabin/NAB-1.

## Results

### Mutants in the Claudin-like Molecule *hic-1* Are Hypersensitive to Aldicarb

We are interested in understanding the function of claudins at the neuromuscular junction (NMJ). In order to study genes that are involved in synaptic functioning at the *C. elegans* NMJ, a behavioral assay (Aldicarb assay) was used. We screened for mutants (Sharma et al., 2018) that were either hypersensitive to Aldicarb (i.e., Hic [hypersensitive to inhibitor of cholinesterase]) or resistant to Aldicarb.

**Figure 1:**
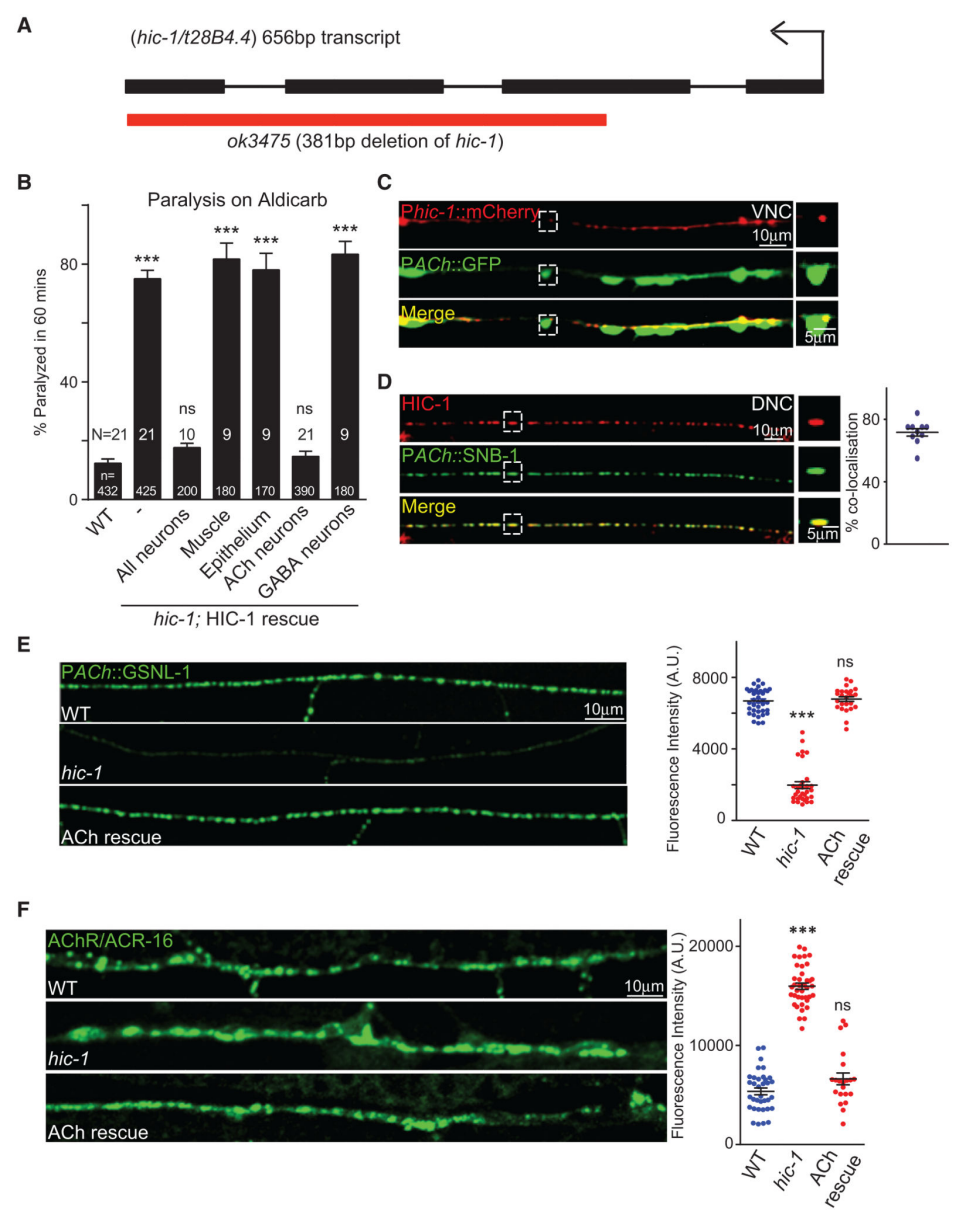
Mutants in *hic-1* Are Hypersensitive to Aldicarb (A) Illustration of the genomic region of *hic-1* introns and exons. The red bar indicates the *hic-1* (*ok3475)* deletion. Also see Figure S1. (B) Percentage paralysis of *C. elegans* at the 60 min time point. Attempted rescue of the Aldicarb phenotype using the following promoters; P*rab-3* (pan-neuronal), P*myo-3* (body-wall muscles), P*let-413* (epithelial cells), P*unc-17* (cholinergic neurons), and P*unc-25* (GABAergic neurons*)*. In all Aldicarb bar graphs, N is the number of trials and n is the total number of animals tested per genotype (~20 animals/trial). (C) Expression of p*hic-1*::mCherry in the ventral nerve cord (VNC) cholinergic neurons that are tagged with GFP. n > 10. Also see Figure S2. (D) The punctate expression of the HIC-1::mCherry overlaps with the SNB-1::GFP at the cholinergic synapses of the dorsal nerve cord (DNC). Percentage co-localization of HIC-1 was calculated using the following formula: (number of HIC-1 puncta co-localized with SNB-1/total number of HIC-1 puncta) × 100 in 100 μm. n = 10. Also see Figure S2. (E) Representative images and quantitation of Gelsolin (GSLN-1)::GFP expressed in a subset of cholinergic neurons. WT (n = 35), *hic-1* (n = 28), and *hic-1;* P*ACh::*HIC-1 (n = 25). (F) Representative images and quantitation of fluorescence intensity of the *C. elegans* NMJ expressing ACR-16::GFP transgene in the body-wall muscles in WT, *hic-1*, and *hic-1*; P*ACh*::HIC-1 animals. n = 35 (WT), n = 40 (*hic-1*), and n = 25 (*hic-1*; P*ACh*::HIC-1). Also see Figure S3. p values were calculated using one-way ANOVA and Bonferroni’s multiple-comparison test. ***p < 0.001. ns, not significant. Data are represented as mean ± SEM.

One of the mutants that was positive from this screen was an as yet uncharacterized protein, T28B4.4/HIC-1. HIC-1 is weakly similar to CLC-1 (claudin-like in *C. elegans*; WormBase). The *hic-1(ok3475)* mutants have a 381 bp deletion in the coding region of the gene that starts in the middle of the second exon and deletes the rest of the gene, indicating that *ok3475* is likely a null allele of *hic-1* (Figure 1A). These mutants of *hic-1* were hypersensitive to Aldicarb (Figure 1B).

The claudin super-family of proteins has four transmembrane domains with their N and C termini inside the cell (Suzuki et al., 2014), which is predicted in HIC-1 (Figure S1A). Most claudins have a PDZ binding motif at the C terminus (reviewed in Simske, 2013), which is conserved in HIC-1 (Figure S1B). Claudins also have two conserved extracellular loops that allow them to make homo- and heterophilic interactions in epithelial cells (Suzuki et al., 2014). The sequence of the first large extracellular loop of HIC-1 showed poor alignment with other claudins (Figure S1C). To check whether HIC-1 shared any similarity to other tetraspan proteins, we analyzed the alignment of HIC-1 amino acid sequences with three tetraspan proteins RYD-2 (Liégeois et al., 2007) and SPE-38 (Chatterjee et al., 2005) in *C. elegans* and CD82 (Hammond et al., 1998) in vertebrates. The HIC-1 sequence did not show presence of any conserved motifs with these proteins (Figure S1D). Taken together, the predicted topology of HIC-1 suggests that HIC-1 could share structural features similar to other members of the claudin superfamily.

Because claudins are required for maintaining the epithelial cell integrity, one concern was that *hic-1* mutants could be compromised at the level of the epithelial cells and hence show increased Aldicarb uptake and paralysis. To see if this was the case, we did two experiments; first, we went on to express HIC-1 in the epithelial cells and found that this did not rescue the Aldicarb hypersensitivity seen in the mutants (Figure 1B), and second, we tested epithelial cell integrity in the mutants using an assay that allows SYTOX green dye uptake. If the epithelial cells are more permeable, as is seen in dead animals, there would be more uptake of SYTOX green (Gill et al., 2003). We found no obvious differences in SYTOX green uptake in *hic-1* mutants in comparison with wild-type (WT) animals (Figure S1E), suggesting that the putative claudin-like protein HIC-1 could be playing a role at the *C. elegans* NMJs and probably does not affect the functioning of epithelial cells.

We next went on to test if the Aldicarb defects of *hic-1* mutants could be attributed to the function of HIC-1 in motor neurons or body-wall muscles. We attempted to rescue the *hic-1*-mutant phenotype in neurons and body-wall muscles using tissue-specific promoters. Pan-neuronal expression of HIC-1 was able to rescue the hypersensitivity to Aldicarb, while body-wall muscle expression of HIC-1 failed to rescue the Aldicarb defects (Figure 1B). We further went on to examine if the expression of HIC-1 in cholinergic or GABAergic neurons, both of which synapse onto the body-wall muscle of *C. elegans* (Alfonso et al., 1993; McIntire et al., 1993a, 1993b) could rescue the Aldicarb phenotype. We found that HIC-1 expression in cholinergic neurons and not GABAergic neurons could rescue the Aldicarb defects seen in the mutants (Figure 1B). These rescue experiments suggest that HIC-1 could function in cholinergic neurons to regulate Aldicarb sensitivity in *C. elegans*.

Because HIC-1 appears to function in cholinergic neurons, we went on to ask if HIC-1 is expressed in these neurons. To determine the expression pattern of HIC-1, we first generated a transcriptional reporter of *hic-1* tagged with mCherry. The *hic-1* promoter expression was seen in the head, in the tail, and along the nerve cord of the animals (Figure S2A). We also observed *hic-1* expression in cholinergic neurons (Figure 1C). To further study the sub-cellular localization of HIC-1, we generated a translational reporter of HIC-1 in which mCherry was cloned under the full-length genomic DNA including the promoter region of HIC-1. We then crossed this translational reporter line with a line expressing the presynaptic marker protein synaptobrevin (SNB-1) which was tagged to GFP and expressed either in cholinergic or in GABA neurons and synapses (Babu et al., 2011; Hao et al., 2012; Sieburth et al., 2005). We detected a co-localization of the translational reporter of HIC-1 with the SNB-1 protein in cholinergic neurons but not in GABA neurons (Figure S2B) and in cholinergic synapses (Figure 1D) but again not in GABAergic synapses (Figure S2C). These data suggest that HIC-1 is localized at the presynaptic termini of cholinergic neurons (Figure 1D). We also looked at the expression of HIC-1 in body-wall muscles and found that HIC-1 did not appear to be expressed in body-wall muscles (Figure S2D). The translational fusion construct of HIC-1 was able to rescue the Aldicarb hypersensitivity that was seen in *hic-1* mutants (Figure S2E), suggesting that the mCherry tag was unlikely to be hampering the function of the gene.

Because HIC-1 appeared to be expressed in cholinergic neurons and synapses, we next went on to see if *hic-1* mutants showed defects in cholinergic neurons and/or synapses. In order to see if HIC-1 is required for the development of neurons or synapses, we initially crossed the *hic-1*-mutant animals into marker lines that showed expression in cholinergic or GABAergic neurons (Babu et al., 2011; Sieburth et al., 2005). We found that the *hic-1* mutants did not show defects in the development of cholinergic or GABAergic neurons (Figure S3A). Furthermore, on crossing the *hic-1* mutants with the active zone marker α-Liprin/SYD-2, expressed specifically in cholinergic or GABAergic synapses (Sieburth et al., 2005; Yeh et al., 2005), we found no defects in neuromuscular synapse development (Figure S3B). Next, we went on to analyze the synaptic vesicle protein SNB-1 (Sieburth et al., 2005) and again found no defects in presynaptic SNB-1 fluorescence in *hic-1* mutants (Figure S3C). We then visualized the actin network at the cholinergic synapses by using an actin-binding protein Gelsolin (GSLN-1) tagged with GFP and expressed in cholinergic neurons and synapses (Sieburth et al., 2005). We observed that the GSLN-1::GFP fluorescence intensity was significantly reduced in the *hic-1* mutants in comparison with WT control animals (Figure 1E). The decreased levels of Gelsolin in *hic-1* were completely rescued by expressing HIC-1 in cholinergic neurons (Figure 1E). These results suggest that the actin cytoskeleton could be disrupted in the absence of *hic-1*.

Taken together, our data so far indicate that at the presynaptic terminal, *hic-1* mutants appear to regulate actin cytoskeleton in the cholinergic neurons. We went on to analyze postsynaptic receptor levels at the NMJ in these mutants.

The *C. elegans* body-wall muscle expresses one GABA and two sets of acetylcholine receptors (AChRs), one that is a nicotinic acetylcholine receptor (nAChR) and made up of homomeric subunits of AChR/ACR-16 and the other that is sensitive to the drug levamisole (LAChR) and consists of heteropentameric αβ subunits (Richmond and Jorgensen, 1999). We first went on to test the two AChRs (Babu et al., 2011; Francis et al., 2005) and found a significant increase in AChR/ACR-16 levels at the NMJ in *hic-1* mutants. This increase was completely rescued by expressing HIC-1 in cholinergic neurons (Figure 1F). Furthermore, to test if the increase in AChR/ACR-16 levels was due to increased expression of AChR/ACR-16, we performed qPCR experiments to quantify the levels of *AChR/acr-16* RNA in WT and *hic-1* mutants and found no differences in the RNA levels of AChR/ACR-16 in *hic-1* mutants (Figure S3G). Testing a subunit of the LAChR/UNC-29 showed no obvious changes in LAChR/UNC-29 levels at the NMJ (Figure S3E). Similarly, there were no significant defects in GABAR/UNC-49 levels in *hic-1* mutants (Figure S3D).

To further probe whether *hic-1* affects AChR/ACR-16, we made double mutants of *hic-1* and *AChR/acr-16*. The double mutants showed a resistant phenotype in Aldicarb that was similar to *AChR/acr-16* single mutants; that is, *AChR/acr-16* mutants appeared to completely suppress the hypersensitivity seen in the *hic-1* mutants (Figure S3F).

### Mutants in *hic-1* Show Aberrant Muscle Responsiveness

**Figure 2:**
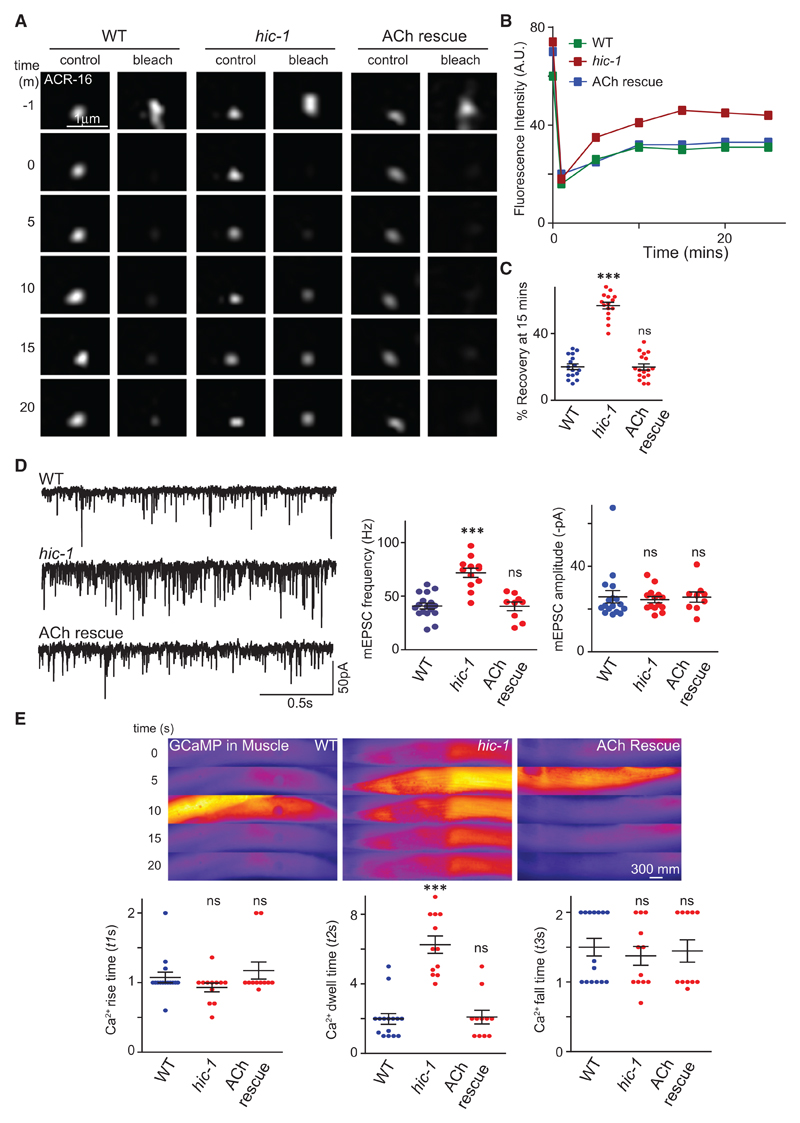
Muscle Responsiveness Is Aberrant in *hic-1*-Mutant Animals (A) Representative time-lapse images at multiple time points of single punctum of ACR-16::GFP; −1 m refers to the time before bleaching. The punctum was photobleached at the 0 min time point. The fluorescence recovery and the percentage of recovery were calculated till the fluorescence intensity reached a plateau (at the 15 min time point). (B) Recovery rate of a single ACR-16::GFP puncta after photobleaching in WT, *hic-1*, and *hic-1*; P*ACh*::HIC-1 animals. (C) Percentage of recovery at the 15 min time point. The number of puncta analyzed and the genotypes tested were n = 15 (WT), n = 18 (*hic-1*), and n = 13 (*hic-1*; P*ACh*::HIC-1). (D) Whole-cell recordings on the muscles were performed to record endogenous acetylcholine (ACh) release (mEPSCs) from WT, *hic-1*, and *hic-1*; P*ACh*::HIC-1 animals. The mEPSC frequency is greater in *hic-1*-mutant *C. elegans* and is rescued by expressing HIC-1 in cholinergic neurons. The mEPSC amplitude was not significantly different across genotypes. The animals tested were n = 17 (WT), n = 12 (*hic-1*), and n = 9 (*hic-1*; P*ACh*::HIC-1). Also see Figure S4A. (E) GCaMP is expressed in the body-wall muscles using a muscle-specific promoter. Representative time-lapse fluorescence images and data analysis of calcium transients in the *C. elegans* muscles are shown here. The *hic-1* mutants show increased calcium transients, which are rescued by expressing HIC-1 in cholinergic neurons. The dot-plot graphs represent rise time (t1s), dwell time (t2s), and fall time (t3s) constants for calcium transients in different genotypes. A representation of the time constants is indicated in Figure S4B. Animals tested: n = 15 (WT), n = 12 (*hic-1*), and n = 11 (*hic-1*;P*ACh*::HIC-1). Also see Figures S4B and S4C. p values were calculated using one-way ANOVA and Bonferroni’s multiple-comparison test. ***p < 0.001; ns, not significant. Data are represented as mean ± SEM.

To further investigate how HIC-1 could be regulating AChR/ACR-16 receptor levels, we performed fluorescence recovery after photobleaching (FRAP) on ACR-16::GFP puncta in WT and *hic-1*-mutant NMJs. FRAP measures diffusion of non-bleached fluorescent proteins to a bleached area (Reits and Neefjes, 2001). The speed and extent of the recovery of the fluorescent protein depends on the movement of the protein. Synapses are thought to have two pools of receptors, one that is immobilized and does not recover after photobleaching and the other that is mobile and comes back to the region of interest (bleached area) with time (Dorsch et al., 2009). Both the percentage and rate of FRAP were significantly higher in *hic-1* mutants (Figures 2A–2C), indicating a higher percentage of mobile fraction of AChR/ACR-16 receptors at the NMJ in *hic-1* mutants. Again, we were able to rescue this phenotype by specifically expressing HIC-1 in cholinergic neurons. These data indicate that HIC-1 could restrict the exchange between synaptic and mobile AChR/ACR-16 receptors by controlling the mobile receptor fraction available for delivery at the synapse.

In order to test if *hic-1* mutants show defects in synaptic physiology, we went on to evaluate synaptic transmission in *hic-1*-mutant animals. We initially measured endogenous postsynaptic currents from NMJs, which reflects synaptic vesicle fusion evoked by the endogenous activity of the motor neurons. The electrophysiology recordings from the muscles showed that the frequency of the miniature excitatory postsynaptic current (mEPSC) was greater in the *hic-1* mutants compared with WT controls (Figure 2D). This increase was rescued by expressing HIC-1 in cholinergic neurons (Figure 2D), but the average amplitude of mEPSCs was comparable with WT animals in *hic-1*-mutant *C. elegans* (Figure 2D). We also measured evoked EPSCs in *hic-1*-mutant animals; the peak amplitude of evoked EPSCs was not significantly different from the WT animals (Figure S4A). These data indicate that HIC-1 affects endogenous but not stimulus-evoked acetylcholine neurotransmitter release at the presynaptic nerve terminals of the NMJ. In addition, the normal amplitudes of the mEPSCs and evoked EPSCs in *hic-1* mutants indicate that the number of functional AChR/ACR-16 receptors at the muscle membrane in *hic-1* is not altered. Because our data indicate that *hic-1* mutants show increased AChR/ACR-16 levels at the NMJ (Figures 1F, S3F, and 2A–2C), this may suggest that the increased AChR/ACR-16 receptors in *hic-1* could be accumulating at non-synaptic or subsynaptic sites.

In order to test if there was any muscle activity defects caused by increased acetylcholine release or increased number of AChR/ACR-16 receptors, we analyzed calcium transients using GCaMP as a transgene expressed in the body-wall muscle of the animals (Schwarz et al., 2012). We measured the duration of the calcium transients in a complete cycle, which is the start of the calcium signals to the maximum peak and then maximum peak to the fall of the transients (Gong et al., 2016). The rise and fall time of the calcium transients in *hic-1* animals was not significantly different from WT animals (Figure 2E), but we observed a significant increase in the dwell time (Figure 2E) and delay in the decay (Δt) of calcium transients in the *hic-1* mutants (Figure S4C). These phenotypes were rescued upon expression of HIC-1 in cholinergic neurons. One possible explanation for these data could be that the increased acetylcholine release from the cholinergic NMJs in *hic-1*-mutant animals (Figure 2D) could lead to increased calcium transients in the *hic-1*-mutant animals. There is also a possibility that the increased subsynaptic fraction of AChR/ACR-16 receptors could also contribute to the increased Calcium response in *hic-1* animals (Brumwell et al., 2002).

We next wanted to investigate the mechanism of HIC-1 function in cholinergic neurons that allow maintenance of normal AChR/ACR-16 levels at the body-wall muscles.

### HIC-1 Is Required to Maintain Normal Wnt Release from Cholinergic Neurons

**Figure 3:**
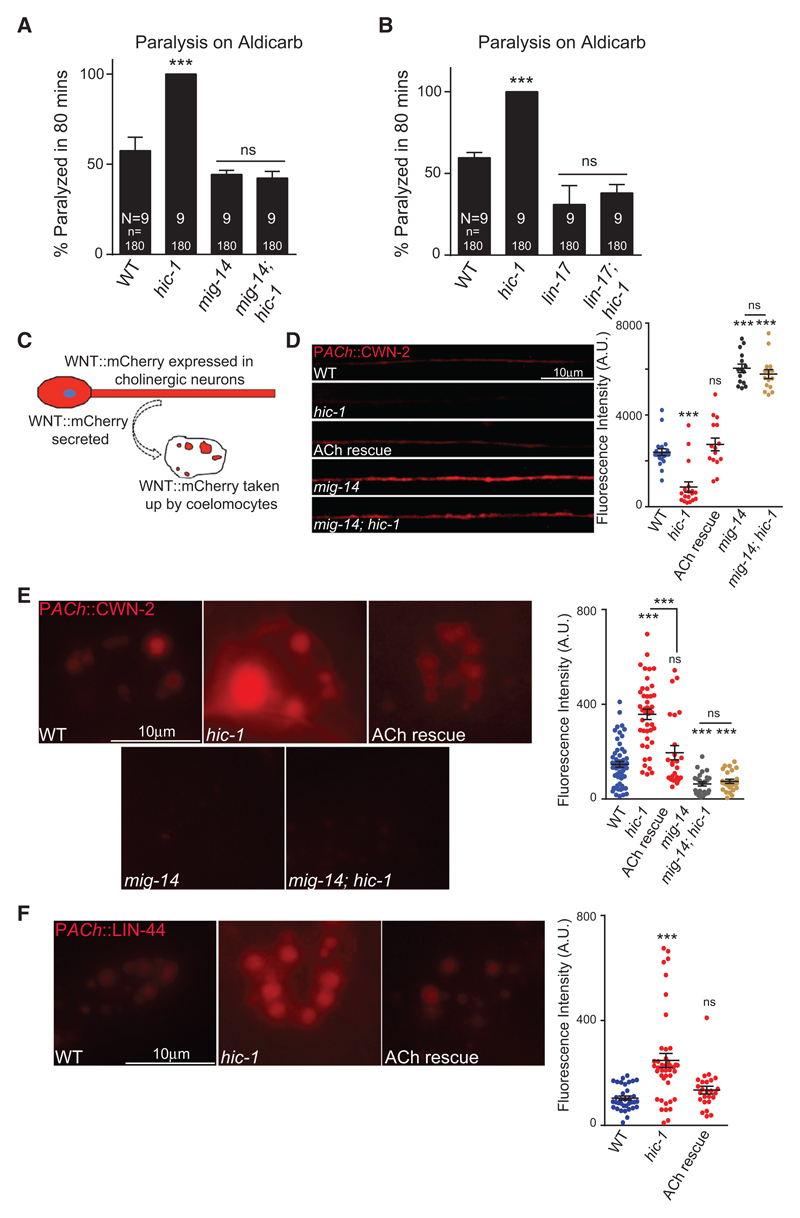
Mutants in *hic-1* Show Increased Wnt Release (A) Aldicarb assay for double mutants containing *wntless/mig-14* and *hic-1* along with control animals. (B) Aldicarb assay for *frizzled/lin-17; hic-1* mutants along with control strains. (C) Illustration of the coelomocyte uptake assay for Wnts. (D) Wnt/CWN-2 fluorescence intensity along the DNC from WT (n = 20), *hic-1* (n = 18), HIC-1 rescue in ACh neurons (n = 15), *mig-14* (n = 15), and *mig-14; hic-1* (n = 15) animals. (E) Representative images and dot plot of coelomocyte fluorescence intensity of Wnt/CWN-2::mCherry in cholinergic neurons from WT (n = 57), *hic-1* (n = 44), P*ACh*::HIC-1 (n = 25), *mig-14* (n = 25), and *mig-14; hic-1* (n = 25) animals. Also see Figure S5. (F) Representative images and quantitation of coelomocyte fluorescence intensity in animals expressing Wnt/LIN-44::mCherry in cholinergic neurons. WT (n = 38), *hic-1* (n = 42), and *hic-1*; P*ACh*::HIC-1 (n = 23). Also see Figure S5. p values were calculated using one-way ANOVA and Bonferroni’s multiple-comparison test. ***p < 0.001; ns, not significant. Data are represented as mean ± SEM.

Our data so far suggest that HIC-1 is functioning in cholinergic motor neurons to regulate the levels of postsynaptic AChR/ACR-16 receptors; we wondered how HIC-1 being a presynaptic molecule could be affecting AChR/ACR-16 levels. Previous studies have suggested that postsynaptic AChR/ACR-16 receptor translocation onto the muscle membrane is regulated by Wnt ligands that are secreted from the presynaptic motor neurons (Babu et al., 2011; Jensen et al., 2012; Pandey et al., 2017). To address the possibility that HIC-1 could be functioning through the Wnt signaling pathway to regulate AChR/ACR-16 delivery at the body-wall muscle, we initially performed Aldicarb assays for different Wnt pathway mutants involved in AChR/ACR-16 regulation in the *hic-1* mutant background. The *C. elegans* Wntless ortholog MIG-14 is required for the secretion of Wnt ligands (Hardin and King, 2008). The *mig-14* mutants were resistant to Aldicarb in comparison with WT animals, while the *mig-14; hic-1* double mutants were able to suppress the hypersensitivity of *hic-1* mutants and show a phenotype indistinguishable from the *mig-14* mutant phenotype (Figure 3A), suggesting that MIG-14 could function downstream of HIC-1. A canonical Wnt ligand, Frizzled/LIN-17, is required for maintaining AChR/ACR-16 in the body-wall muscles (Jensen et al., 2012). We went on to perform Aldicarb assay with *frizzled/lin-17; hic-1* double mutants. Mutants in *frizzled/lin-17* are also resistant to Aldicarb, and the hypersensitivity on Aldicarb that was seen in *hic-1* mutants was completely suppressed by mutants in *frizzled/lin-17* (Figure 3B). Our results so far indicate that HIC-1 could function upstream of the Wnt signaling pathway.

We next wanted to understand how HIC-1 could be affecting the Wnt pathway through its expression in presynaptic neurons. One possible function for HIC-1 could be to regulate Wnt vesicle release from cholinergic synapses that are known to express Wnt (Jensen et al., 2012; Zhang et al., 2012). We went on to perform experiments to check whether HIC-1 was required for Wnt secretion.

To visualize Wnt secretion, we took advantage of the well-established coelomocyte assay in *C. elegans.* Coelomocytes are specialized scavenger cells in *C. elegans*, which take up molecules that are secreted in the body cavity or pseudocoelom (Fares and Greenwald, 2001; Sieburth et al., 2007). Previous reports have also used this assay to study Wnt/CWN-2 and Wnt/LIN-44 secretion (Jensen et al., 2012; Pandey et al., 2017) (Figure 3C). Because both Wnt/CWN-2 and Wnt/LIN-44 have been reported to be required for maintaining AChR/ACR-16 levels at the NMJ, we assayed the secretion of these two Wnts. Wnts tagged with mCherry were expressed in cholinergic neurons and imaged for fluorescence intensity in the dorsal cord as well as in coelomocytes (Figures 3D–3F and S5A). Similar to what is seen with neuropeptides, the dorsal cord punctal fluorescence could correspond to secretory vesicles containing Wnts in the axons, and the coelomocyte fluorescence could indicate Wnts that are secreted from the neurons and accumulate in the coelomocytes (Fares and Greenwald, 2001; Sieburth et al., 2007). We observed that in *hic-1* mutants, coelomocyte fluorescence was significantly increased, whereas the punctal axonal fluorescence intensity of CWN-2 was significantly reduced (Figures 3D, 3E, and S5A). Both the dorsal cord punctal intensity and coelomocyte fluorescence defects of *hic-1* were rescued by expressing HIC-1 in cholinergic neurons (Figures 3D, 3E, and S5A). A similar increase in coelomocyte fluorescence was obtained with the other Wnt (LIN-44) that was tested (Figures 3F and S5A). These data suggest that HIC-1 could be regulating the release of Wnt ligands from cholinergic neurons. In order to confirm that we were indeed looking at Wnt secretion in coelomocytes, we performed two experiments. First, we crossed Wnt-expressing animals with a coelomocyte marker line, P*unc-122*::GFP (Frøkjaer-Jensen et al., 2008). The expression of Wnt endosomes can be seen within the coelomocytes labeled with p*unc-122*::GFP marker in the Wnt-expressing lines (Figure S5B). Second, we analyzed Wnt secretion in the *Wntless*/*mig-14* background. Wntless/MIG-14 is required for the binding and secretion of Wnt ligands from Wnt-producing cells (Eisenmann and Kim, 2000; Thorpe et al., 1997). We observed a significant reduction of Wnt uptake in the coelomocytes and a concomitant increase in the axonal punctal fluorescence in the *Wntless/mig-14* mutants (Figures 3D, 3E, and S5A). In addition, *Wntless/mig-14* suppressed both the increased coelomocyte fluorescence and decreased axonal punctal fluorescence defects of *hic-1* mutants (Figures 3D, 3E, and S5A). To test if HIC-1 could be affecting expression of Wnt ligands, we performed real-time qPCR for the Wnts CWN-2 and LIN-44 in WT, *hic-1*, and *Wntless*/*mig-14* mutant animals. No significant difference in the RNA levels of CWN-2 and LIN-44 in either of the two mutants in comparison with WT animals was detected (Figure S5C), indicating that HIC-1 does not appear to affect the expression of Wnt genes.

Because HIC-1 appears to be affecting Wnt release from cholinergic neurons, we next asked if HIC-1 could function as a more general molecule involved in the release of small proteins from cholinergic neurons. To this end we performed two experiments; first we performed an Aldicarb assay to see if mutants in *hic-1* could function in the same pathway as neuropeptides. We tested the Aldicarb phenotype of *egl-21* (a carboxypeptidase required for the normal synthesis of neuropeptides); in accordance with previous work, we also found that *egl-21* mutants were resistant to Aldicarb (Jacob and Kaplan, 2003), while the *egl-21; hic-1* double mutants showed a phenotype that was intermediate between the resistance seen in *egl-21* mutants and the hypersensitivity to Aldicarb seen in *hic-1* mutants (Figure S5D). These data indicate that HIC-1 may not be functioning through neuropeptides. Next, we went on to look at the expression of the neuropeptide NLP-21 tagged with YFP and expressed in cholinergic neurons (Staab et al., 2013) in WT and *hic-1* mutants. On imaging the dorsal cord and the coelomocytes in these mutants, we saw no significant differences in the fluorescence intensity in either the cord or the coelomocytes (Figures S5E and S5F). Again we found no change in RNA levels of NLP-21 in *hic-1* mutants (Figure S5C). These data indicate that *hic-1* does not appear to affect neuropeptide secretion from cholinergic synapses, indicating that HIC-1 is not a general factor affecting constitutive secretion of small molecules.

Taken together, these data indicate that the HIC-1 is required for normal Wnt secretion from cholinergic neurons and that HIC-1 functions upstream of the Wnt signaling pathway to regulate AChR/ACR-16 levels at the NMJ. We next wanted to understand how HIC-1 could be affecting Wnt secretion.

### HIC-1 Is Required to Maintain the Actin Cytoskeleton in Cholinergic Neurons

**Figure 4:**
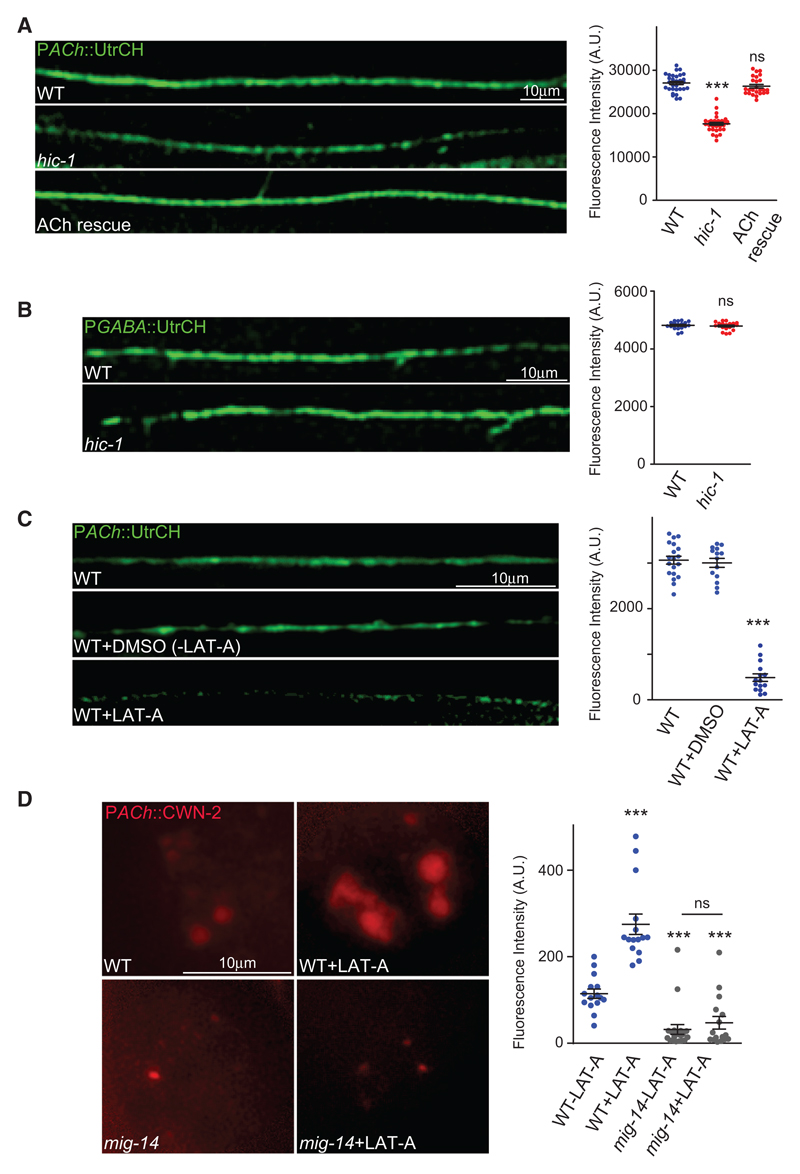
HIC-1 Is Required to Maintain a Normal Actin Cytoskeleton at Cholinergic Synapses (A) Representative images and quantitation of P*ACh*::GFP-UtrCH fluorescence intensity along the DNC of *C. elegans*. WT (n = 30), *hic-1* (n = 27), P*ACh*::HIC-1 (n = 23). (B) Representative images of the DNC and quantitation for P*GABA::*GFP-UtrCH in WT (n = 15) and *hic-1* (n = 17) mutant animals. (C) Representative images and quantitation of the DNC of animals expressing P*ACh*::GFP-UtrCH. WT (n = 19) and WT animals injected with DMSO (n = 14) or DMSO and latrunculin-A (LAT-A) (n = 15) were imaged for this experiment. (D) Representative images and quantitation of coelomocyte fluorescence in WT and *mig-14* strains that express P*ACh*::Wnt/CWN-2::mCherry. WT injected with DMSO (n = 20), WT injected with LAT-A (n = 15), *mig-14* injected with DMSO (n = 20), and *mig-14* injected with LAT-A (n = 16). In (A), (C), and (D), p values were calculated using one-way ANOVA and Bonferroni’s multiple-comparison test. In (B), p values were calculated using two-tailed unpaired Student’s t test. ***p < 0.001; ns, not significant. Data are represented as mean ± SEM.

Claudins interact with the actin cytoskeleton via mediator proteins (Kojima et al., 2013). We were interested in testing if HIC-1, as a possible claudin-like protein, could be involved in maintaining the actin cytoskeleton. As shown previously, HIC-1 is required to maintain the normal levels of the actin-binding protein Gelsolin in the cholinergic neurons (Figure 1E). To further consolidate these results, we visualized stable F-actin cytoskeleton in cholinergic synapses using an actin-binding probe, utrophin (Burkel et al., 2007), tagged with GFP at its N terminus (GFP-UtrCH) expressed in the cholinergic neurons. A decrease in GFP-UtrCH intensity was observed with cholinergic GFP-UtrCH in *hic-1* mutants, and this phenotype was rescued by expressing HIC-1 in cholinergic synapses (Figure 4A). As a control experiment, we examined GFP-UtrCH fluorescence in GABA synapses and found this fluorescence to be unaltered (Figure 4B). These data indicate that HIC-1 is involved in maintaining the actin cytoskeleton network specifically at cholinergic synapses.

We next wondered if HIC-1 could be affecting Wnt release by regulating the actin cytoskeleton. To address this possibility, we disrupted the F-actin cytoskeleton by injecting WT animals with latrunculin A (Coué et al., 1987). Initially we confirmed that LAT-A indeed disrupted the F-actin cytoskeleton, as seen by visualizing GFP-UtrCH in LAT-A treated, untreated, and control-treated animals (Figure 4C). Next, we imaged the Wnt ligand CWN-2 tagged with mCherry in the coelomocytes. We found a significant increase in CWN-2::mCherry fluorescence in the coelomocytes of LAT-A-treated animals in comparison with mock-treated *C. elegans* (Figure 4D). Furthermore, the coelomocyte fluorescence was significantly reduced in *mig-14* animals after LAT-A treatment (Figure 4D). Together, these data suggest that depolymerization of the F-actin cytoskeleton may cause increased Wnt release, indicating that the F-actin cytoskeleton could be acting as a “brake” for Wnt vesicle release and disrupting F-actin could cause an uncontrolled release of Wnt ligands (Figure 4D).

### HIC-1 Interacts with an Actin-Binding Protein, Neurabin

**Figure 5:**
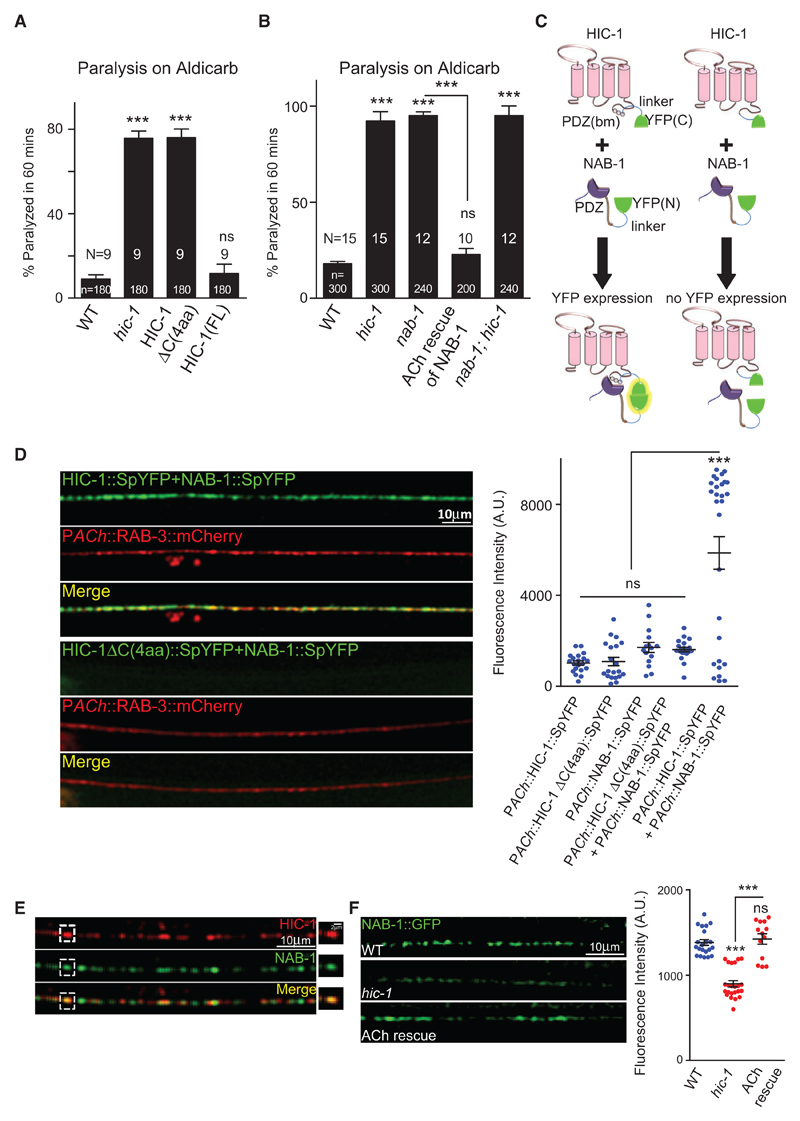
HIC-1 Interacts with Neurabin through Its PDZ(bm) (A) Percentage paralysis of *C. elegans* at 60 min after Aldicarb exposure. HIC-1*Δ*C(4aa) indicates a deletion of the last four amino acids from C terminus of HIC-1. Also see Figure S6A. (B) Percentage paralysis of *C. elegans* at 60 min after Aldicarb exposure indicating genetic interaction between *hic-1* and *nab-1.* Also see Figure S6B. (C) Schematic indicating possible results of the BiFC assay between HIC-1 and NAB-1. HIC-1 (pink) is tagged with the C-terminal half of YFP (green) via a linker sequence (blue), the PDZ(bm) is indicated as circles. The C terminus of NAB-1 is tagged to the N-terminal half of YFP (green) using a linker sequence (blue). The interaction between NAB-1 and HIC-1 leads to reconstitution of YFP fluorescence (yellow glow), while no fluorescence is detected in the absence of the PDZ(bm) of HIC-1. (D) Representative images and quantification of the DNC of WT animals expressing either HIC-1::SpYFP and NAB-1::SpYFP together or HIC-1*Δ*C(4aa) and NAB-1::SpYFP together in the cholinergic neurons. The cholinergic synapses are labeled with RAB-3::mCherry. Right: quantification of the YFP reconstitution between HIC-1 and NAB-1 along with multiple controls. Also see Figures S6C and S6D. (E) Representative image of the DNC of *C. elegans* expressing HIC-1::mCherry and NAB-1:GFP. Partial co-localization was seen for HIC-1 and NAB-1 (n > 10). (F) Representative images and quantitation of NAB-1::GFP fluorescence intensity along the DNC in WT (n = 21), *hic-1* (n = 23), *and hic-1;* P*ACh::*HIC-1 (n = 13) animals. Also see Figure S6E. p values were calculated using one-way ANOVA and Bonferroni’s multiple-comparison test. ***p < 0.001; ns, not significant. Data are represented as mean ± SEM.

Next, we wanted to investigate the interacting partner(s) of HIC-1 at the synapse. To test if the PDZ binding motif [PDZ(bm)] of HIC-1 is involved in the function of HIC-1 at the NMJ, we deleted the last four amino acids of the HIC-1 protein, which is the putative PDZ(bm), and expressed this protein (HIC-1ΔC(4aa)) under the cholinergic promoter in *hic-1* mutants. The truncated protein failed to rescue the hypersensitivity phenotype seen in the *hic-1*-mutant animals (Figure 5A), suggesting that the putative PDZ(bm) is likely to be required for HIC-1’s binding to mediator protein(s) that may help in the association of HIC-1 to the actin cytoskeleton. To test if the putative PDZ(bm) of HIC-1 is required for the synaptic localization of the protein, we visualized the HIC-1ΔC(4aa) truncated protein at the synapse by tagging this deleted protein with mCherry; the HIC-1ΔC(4aa) was co-localized with SNB-1 in the cholinergic neurons, indicating that this protein showed normal localization at the synapse, similar to that seen in the control FL HIC-1 (Figure S6A).

Next, we went on to find a possible interactor of HIC-1. Because HIC-1 does not have an actin-binding domain, we decided to search for putative HIC-1-interacting proteins by searching for proteins that satisfied the following criteria: (1) should be present at the synapse, (2) should have an actin-binding domain, and (3) should also have a PDZ domain. While searching through the literature and WormBase, we found one such protein, Neurabin/NAB-1, that fulfilled all the above criteria. NAB-1 is required for instructing synapse assembly by linking adhesion molecules and F-actin to active zone proteins (Chia et al., 2012). We asked if NAB-1 could interact with HIC-1 and thus act as an adaptor to link HIC-1 with F-actin.

We initially performed Aldicarb assays for *nab-1* and found that *nab-1* animals were hypersensitive to Aldicarb (Figure 5B). The hypersensitivity seen in *nab-1* mutants was rescued by expressing NAB-1 under its own promoter in a translational reporter line tagged to GFP (Figure S6E) and by expressing NAB-1 specifically in cholinergic neurons (Figures 5B and S6B). The mutants of *nab-1; hic-1* also showed hypersensitivity toward Aldicarb similar to that seen in *nab-1* or *hic-1* single mutants (Figures 5B and S6B). These data suggest that *nab-1* genetically interacts with *hic-1*. Next, we performed a split YFP/BiFC experiment to explore the possibility of a direct interaction between HIC-1 and NAB-1. Bimolecular fluorescence complementation (BiFC) analysis enables direct visualization of protein interactions by measuring the association of two non-fluorescent fragments of a fluorescent protein fused to putative interacting partners (Kerppola, 2013).

A bright YFP fluorescence was detected at the cholinergic synapses labeled with RAB-3::mCherry (Petrash et al., 2013), when HIC-1 tagged with the C terminus half of YFP and NAB-1 tagged with the N terminus half of YFP were coinjected in the animals (Figures 5C and 5D), whereas YFP fluorescence intensity was significantly reduced when the HIC-1ΔC(4aa)::SpYFP and NAB-1::SpYFP were used in the above experiment (Figures 5C and 5D). The controls, HIC-1::SpYFP, HIC-1ΔC(4aa)::SpYFP, and NAB-1::SpYFP, also showed a significantly reduced YFP signal at the cholinergic synapse (Figure S6D), indicating that the interaction/YFP signal that was seen with HIC-1::SpYFP and NAB-1::SpYFP was likely because of an interaction between HIC-1 and NAB-1. Furthermore, the HIC-1::SpYFP but not the HIC-1ΔC(4aa)::SpYFP was able to rescue the Aldicarb hypersensitivity defect of *hic-1*-mutant animals, and the NAB-1:: SpYFP also rescued the hypersensitivity to Aldicarb seen in the *nab-1* mutants (Figure S6C).

To further investigate the interaction between NAB-1 and HIC-1, we looked at the localization of NAB-1 and HIC-1 using the P*hic-1*::HIC-1::mCherry and the P*nab-1*::NAB-1::GFP lines (Hung et al., 2007) and found a partial co-localization between the translational reporters at the synapse (Figure 5E). If HIC-1 is modulating the F-actin cytoskeleton via NAB-1, we hypothesized that NAB-1 expression at the synapse could be dependent on HIC-1. In order to test this, we analyzed the localization of NAB-1 in *hic-1* animals and found that the NAB-1::GFP fluorescence intensity was significantly reduced in *hic-1* mutants. This phenotype was rescued by expressing HIC-1 in cholinergic neurons in these *C. elegans* (Figure 5F).

Taken together, these data suggest that HIC-1 interacts with NAB-1 through its PDZ(bm) and that NAB-1 localization is dependent on HIC-1 expression.

### HIC-1 and NAB-1 Function in the Same Signaling Pathway

**Figure 6:**
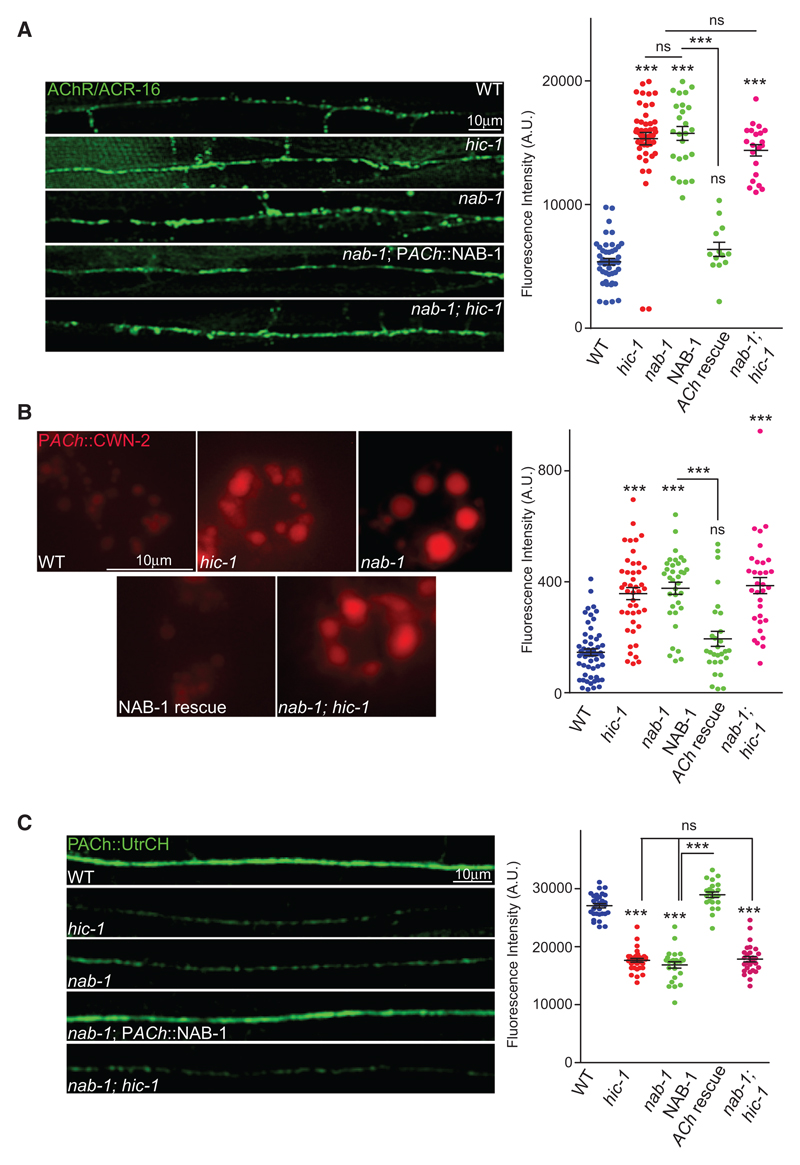
HIC-1 and Neurabin Function Together for Normal Wnt Release (A) Representative images and quantitation of fluorescence intensity along the DNC in animals expressing P*myo-3::*ACR-16::GFP.WT (n = 45), *hic-1* (n = 48), *nab-1* (n = 25), *nab-1;* P*ACh::NAB-1* (n = 18), and *nab-1; hic-1* (n = 21). (B) Representative images and quantitation of coelomocyte fluorescence intensity from P*ACh*::Wnt/CWN-2::mCherry. WT (n = 57), *hic-1* (n = 44), *nab-1* (n = 35), *nab-1*; P*ACh*::NAB-1 (n = 28), and *nab-1; hic-1* (n = 32). Also see Figure S7A. (C) Representative images and quantitation of DNC fluorescence from P*ACh*::GFP-UtrCH expressing animals. WT (n = 25), *hic-1* (n = 20), *nab-1* (n = 20), *nab-1*; P*ACh*::NAB-1 (n = 21), and *nab-1; hic-1* (n = 25). p values were calculated using one-way ANOVA and Bonferroni’s multiple-comparison test. ***p < 0.001; ns, not significant. Data are represented as mean ± SEM.

To further validate the interaction between NAB-1 and HIC-1, we performed further experiments to see if *nab-1* mutants showed phenotypes similar to that seen with *hic-1*. We looked at the ACR-16::GFP, CWN-2::mCherry, LIN-44, and GFP-UtrCH levels in *nab-1* mutants. The *nab-1* mutants showed similar phenotypes as *hic-1* mutants in these experiments, and the nab-1 phenotype was rescued by expressing NAB-1 in cholinergic neurons (*nab-1*; P*ACh*::NAB-1) (Figures 6A–6C and S7A). Furthermore, the double mutants *nab-1;hic-1* also behaved similarly to the single mutants *nab-1* and *hic-1* in these experiments. Taken together, these experiments point toward a direct interaction between HIC-1 and NAB-1, where they are both involved in a signaling pathway mediating normal Wnt secretion and maintenance of the actin cytoskeleton at cholinergic synapses.

### HIC-1 with a C-Terminal NAB-1(ABD) Is Sufficient to Rescue the Wnt Release Defects Associated with *nab-1; hic-1* Double Mutants

**Figure 7:**
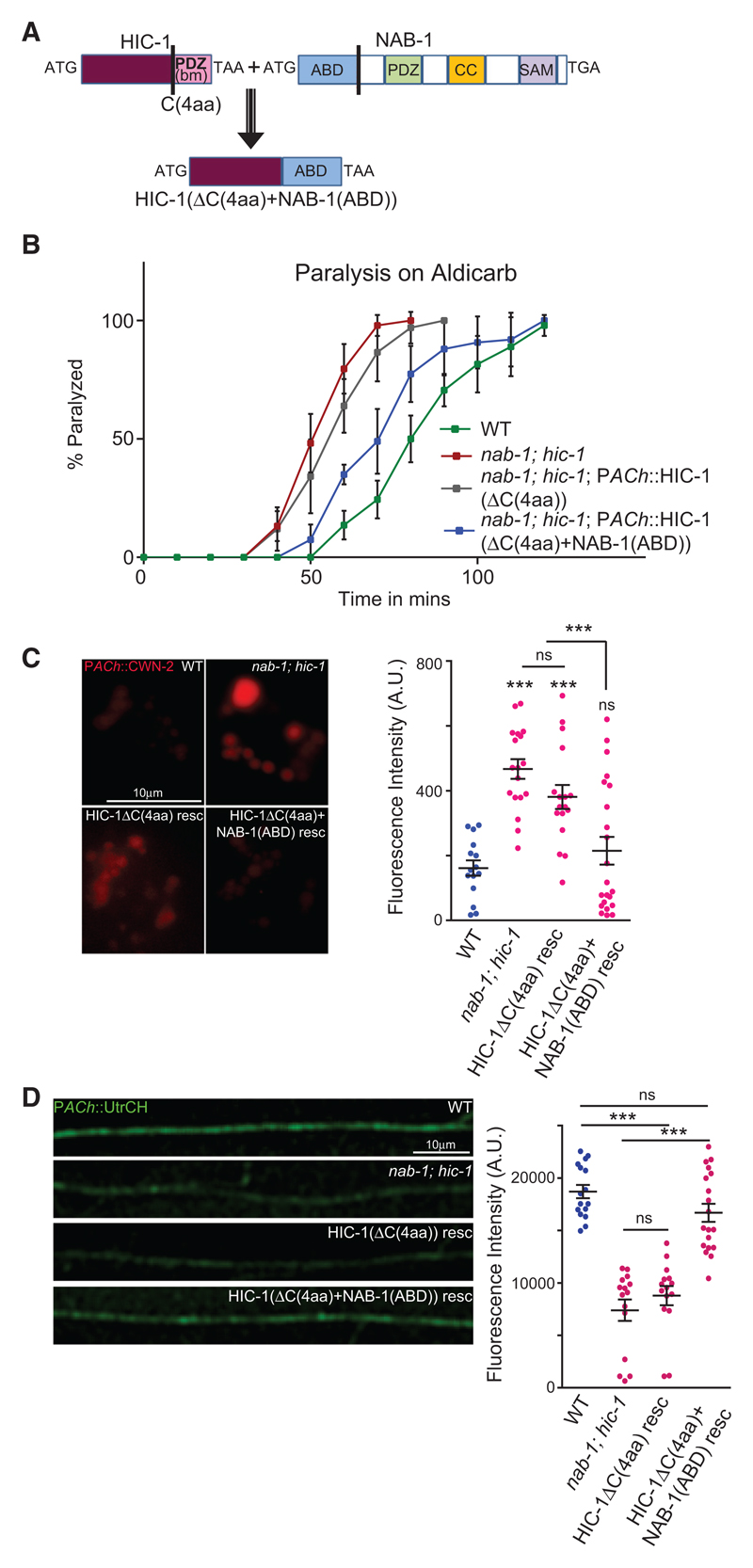
The ABD of Neurabin Linked to HIC-1 Is Sufficient to Rescue the NMJ Defects of the *nab-1; hic-1* Double Mutants (A) Schematic of the fusion construct HIC-1(ΔC(4aa)+NAB-1(ABD)). The C-terminal four amino acids were deleted from HIC-1, and the ABD of NAB-1 was added in frame with the above HIC-1 construct. The domains of NAB-1 are shown (Chia et al., 2012). (B) Time course paralysis on Aldicarb for the following strains: WT, *nab-1; hic-1*, *nab-1; hic-1*; P*ACh*::HIC-1(ΔC(4aa)), and *nab-1; hic-1*; P*ACh*::HIC-1(ΔC (4aa)+NAB-1(ABD)). The experiment is performed six times, and the total number of animals used in each experiment was 120 (20 animals/trial) for each genotype. (C) Representative images and quantitation of coelomocyte fluorescence intensity from P*ACh*::Wnt/CWN-2::mCherry. WT (n = 15), *nab-1;hic-1* (n = 18), *nab-1; hic-1*; P*ACh*::HIC-1(ΔC(4aa)) (n = 17), and *nab-1; hic-1*; P*ACh*::HIC-1(ΔC(4aa)+NAB-1(ABD)) (n = 22). (D) Representative images and quantitation of the DNC of animals expressing GFP-UtrCH as a transgene in cholinergic neurons. The genotypes used in this experiment were WT (n = 16), *nab-1; hic-1* (n = 15), *nab-1; hic-1*; P*ACh*::HIC-1(ΔC(4aa)) (n = 15), and *nab-1; hic-1*; P*ACh*::HIC-1(ΔC(4aa)+NAB-1(ABD)) (n = 19). In (C) and (D), p values were calculated using one-way ANOVA and Bonferroni’s multiple-comparison test. ***p < 0.001; ns, not significant. Data are represented as mean ± SEM.

To further assert the role of HIC-1 through the NAB-1(ABD) in maintaining Wnt release and a normal F-actin cytoskeleton at the NMJ, we made a construct that removed the PDZ-interacting amino acids of HIC-1 and replaced it with the actin-binding domain (ABD) of NAB-1 (HIC-1(ΔC(4aa)+NAB-1(ABD)); Figure 7A). We then went on to test the rescuing activity of this construct in the *nab-1; hic-1* double mutants. We found that in comparison with HIC-1ΔC(4aa), which did not rescue the hypersensitivity to Aldicarb seen in the *nab-1; hic-1*-mutant animals, the HIC-1ΔC(4aa)+NAB-1(ABD) construct partially rescued the hypersensitivity to Aldicarb seen in these animals (Figure 7B). These data indicate that the function of HIC-1 in maintaining normal Aldicarb sensitivity could be largely due to HIC-1 interacting with NAB-1 and allowing the maintenance of a normal actin cytoskeleton and hence normal Wnt release.

We went on to test the rescue of defects in Wnt release and the F-actin cytoskeleton in the *nab-1; hic-1* double-mutant animals. We found that again, while the HIC-1ΔC(4aa)-expressing line could not rescue the Wnt/CWN-2 release defects associated with the *nab-1; hic-1* mutants, the HIC-1ΔC(4aa)+NAB-1(ABD)-expressing animals could partially rescue the Wnt/CWN-2 release defects seen in the mutants (Figure 7C). Finally, we visualized the actin cytoskeleton using GFP-UtrCH expressed in cholinergic neurons in *nab-1; hic-1* animals and the double mutant animals expressing the chimeric protein HIC-1 (ΔC(4aa)+NAB-1(ABD)) or the deletion HIC-1ΔC(4aa), specifically in cholinergic neurons. Consistent with our previous results, we again found that only the chimera of HIC-1 and NAB-1(ABD) could rescue the F-actin defect in the *nab-1; hic-1* mutants.

Together, these data indicate that the ABD of NAB-1 anchored to HIC-1 is sufficient to rescue the NMJ defects of increased Wnt secretion seen in the double mutants of *hic-1* and *nab-1*.

## Discussion

### HIC-1 and NAB-1 Are Regulators of Wnt Secretion

Wnt secretory molecules are known to enable synaptic communication through their function in both pre- and postsynaptic compartments. Jensen et al. (2012) elegantly dissected the role of the presynaptic Wnt/CWN-2 and its receptor Frizzled/LIN-17 in the translocation of AChR/ACR-16 receptors on to the muscle membrane at the *C. elegans* NMJ. Data from the coelomocyte uptake assay indicates that the release of Wnt/CWN-2 and Wnt/LIN-44 ligands from the motor neurons in *hic-1*, *nab-1*, and *nab-1; hic-1*-mutant animals occurs in an uncontrolled manner in comparison with WT control animals, although the initial levels of Wnts were likely the same, as indicated by no change in RNA levels in these mutants. These data potentially indicate the characterization of a loss-of-function mutant that causes an increase in Wnt secretion. More comprehensive studies on how Wnt secretion is deregulated in such mutants could be helpful in treating illnesses in which blocking Wnt secretion or designing therapeutic targets against Wnt signaling have proved helpful (Wang et al., 2016a, 2016b).

Neurabin/NAB-1 is a multidomain protein that plays diverse functions in the nervous system. It has an F-actin binding domain at the N terminus, a PDZ domain that is reported to bind to transmembrane proteins, and a coiled-coil domain at its C terminus (Nakanishi et al., 1997). The specificity-determining CAMs SYG-1 and SYG-2 locally assemble F-actin in *C. elegans* HSN neurons, and NAB-1 then binds to F-actin and goes on to recruit active zone proteins SYD-1 and SYD-2 (Chia et al., 2012). These data point to the fact that NAB-1 could have multiple roles in the nervous system, and its interaction with HIC-1 to allow normal Wnt secretion is probably just one of the many processes it could be involved in. One could imagine that it may function as an adaptor for multiple proteins to allow interaction of different proteins with F-actin and hence regulate different processes in the nervous system.

### Role of the Actin Cytoskeleton in Presynaptic Release

The actin cytoskeleton is involved in myriad processes at the nerve terminals ranging from neurogenesis to axon branching, cellular trafficking and signaling, synaptic vesicles release, and synaptogenesis among others (Morales et al., 2000; Zhang and Benson, 2002). Interestingly, conflicting reports can be found on the role of local F-actin with respect to neurotransmitter release. An earlier report claims that depolymerization of F-actin blocks neurotransmitter release (Bernstein and Bamburg, 1989), while more recent studies suggest an increase in neurotransmitter release upon actin depolymerization (Morales et al., 2000). Morales et al. (2000) found that the depolymerization of actin by latrunculin A treatment transiently increased neurotransmitter release in cultured hippocampal neurons. Our results indicate that HIC-1 is required to maintain the presynaptic actin cytoskeleton and acetylcholine release from the cholinergic neurons in *C. elegans*. We hypothesize that HIC-1 might be regulating acetylcholine release through maintaining a stable F-actin in cholinergic neurons because, in the absence of HIC-1, we see disrupted F-actin, which could lead to increased acetylcholine release. These findings warrant more studies to pinpoint the role of the actin cytoskeleton in neurotransmitter release.

We also see increased Wnt secretion in *hic-1* mutants, which prompted us to ask whether there was any correlation between a normal actin cytoskeleton and Wnt release. Our experiments indicate that disrupting the actin cytoskeleton allows increased Wnt secretion from the cholinergic motor neurons. How the actin cytoskeleton is involved in Wnt release is an important question that would require further experimentation.

For a very long time, Wnts were considered to be developmental molecules regulating embryonic development and early pattering of the embryo (reviewed in Cadigan and Nusse, 1997; Logan and Nusse, 2004). Recent studies highlight more diverse functions of the Wnt signaling in the development and function of synapses and more specifically in maintaining AChR levels at the synapse (Babu et al., 2011; Barik et al., 2014; Henriquez et al., 2008; Jensen et al., 2012; Kamimura et al., 2013; Klassen and Shen, 2007; Messéant et al., 2017; Pandey et al., 2017).

Despite the knowledge we have so far on the role of Wnts in the normal functioning of NMJs, there is no clear experimental evidence implicating the role of F-actin in Wnt secretion from neurons. Koles and Budnik (2012a) described the cellular machinery used in the release of Wnts from the *Drosophila* larval NMJs. They propose that Wnt/Wg is encapsulated in exosomes in conjunction with Wntless/Evi (MIG-14 in *C. elegans*), then Wnt/Wg containing exosomes are sorted into multivesicular bodies at the presynaptic termini; these multivesicular bodies are then fused to the presynaptic membrane at a site near the active zone (the periactive zone). They then release their exosomal content into the synaptic cleft at this site. On the basis of previous reports and our findings, we are proposing that HIC-1 is present at the presynaptic terminal of cholinergic neurons, where it regulates local F-actin dynamics, which in turn allows for normal secretion of both acetylcholine neurotransmitter and Wnt vesicles. An in-depth knowledge of the functioning of the actin cytoskeleton and its involvement in secretion warrants further studies.

### The Intracellular C-Terminal Region of HIC-1 Acts as a Claudin at the NMJ

Out of 23 claudins in humans, 9 have been reported to have a conserved tyrosine residue at their C terminus. It has been shown that modifications to this tyrosine residue (phosphorylation) can alter the affinity of claudin-1 and claudin-2 toward the N-terminal PDZ domain of the ZO-1 protein (Nomme et al., 2015). We found the presence of a tyrosine residue in HIC-1 at the conserved C-terminal site similar to what is seen in other claudins. Hence, we reasoned that HIC-1 could have a putative PDZ(bm) at its C terminus. Furthermore, claudins interact with cytoplasmic scaffolding proteins, particularly ZO-1,2,3, which indirectly connect claudins to the actin cytoskeleton and thereby stabilize tight junction assembly and their barrier functions (Umeda et al., 2006). A deletion construct of HIC-1 that removed the putative PDZ(bm) failed to rescue the Aldicarb defects of HIC-1 and was not able to bind to the PDZ domain of the actin-binding protein Neurabin. Together these data indicate that HIC-1 could be behaving in a manner similar to other bona fide claudins intracellularly, using its C-terminal PDZ(bm) (Figure S7B). Apart from HIC-1 we have recently shown that a claudin-like molecule, HPO-30, is required to maintain levamisole-sensitive AChRs at the NMJ (Sharma et al., 2018). HPO-30 has also been shown to allow normal actin assembly and dendritic branching through its interaction with the WAVE regulatory complex in *C. elegans* (Zou et al., 2018). These results indicate that understanding the function of claudins at synapses and neurons could be essential for us to get insight into the molecules involved in maintaining the actin cytoskeleton in the nervous system.

Claudins are known to make homo- or heterophilic interactions via their two highly conserved extracellular loops; we found these loops in HIC-1 to be poorly aligned with other claudins. This gives rise to the possibility that HIC-1 may not be a traditional claudin or claudin-like molecule and suggests that HIC-1 might not be functioning similarly to other claudins extracellularly. More studies are needed to understand how HIC-1 might be functioning extracellularly and whether it is involved in making *cis*- or *trans*-interactions with other claudins, to other synaptic adhesion molecules or ligands present in the synaptic cleft.

### Implications of the Putative Claudin-like Molecule HIC-1 in Understanding Claudin Functions in the Mammals

Differential expression and function of claudins in mammals has been reported in many tissue (reviewed in Krause et al., 2008a). Apart from the canonical roles of claudins in maintaining barrier functions, they also play diverse non-canonical functions in cell signaling (Hagen, 2017). They make up essential components of the blood-brain barrier, and their deregulation is associated with various brain disorders (reviewed in Gonçalves et al., 2013). Despite their various functions in the brain, the underlying mechanism of claudin function in the brain and at the synapse is yet largely unknown. Our study describes the functional roles for a putative claudin-like molecule, HIC-1, at *C. elegans* NMJs. The findings from this study could help in further understanding the function of claudins and other tetraspan proteins in the brain.

## STAR★METHODS

### Key Resources Table

**Table:** 

REAGENT or RESOURCE	SOURCE	IDENTIFIER
Bacterial and Virus Strains		
*Escherichia coli*, OP50	*Caenorhabditis* Genetics Center	WormBase ID: OP50
*Escherichia coli*, DH5α	ThermoFisher Scientific	Cat#8265017
*Escherichia coli*, DH5β	NEB	Cat#C30191
Chemicals, Peptides, and Recombinant Proteins		
Aldicarb	Sigma	Cat#33386
Sytox green	Sigma	Cat#S7020
Latrunculin A	Sigma	Cat#L5163
Critical Commercial Assays		
cDNA synthesis kit	Roche	Cat#04897030001
SYBR Premix Ex TaqII master mix	Clontech	Cat#RR820B
RNeasy Plus Mini Kit	QIAGEN	Cat#74136
Experimental Models: Organisms/Strains		
N2 *C.elegans* wild isolate	*Caenorhabditis* Genetics Center	WormBase ID: N2
Mutant *hic-1(ok3475) X*	*Caenorhabditis* Genetics Center	Strain RB2512
Mutant *nab-1(ok943) I*	*Caenorhabditis* Genetics Center	Strain RB1017
Mutant *acr-16(ok789) V*	*Caenorhabditis* Genetics Center	Strain RB918
Mutant *mig-14(mu71) II*	*Caenorhabditis* Genetics Center	Strain CF367
Mutant *lin-17(n671) I*	*Caenorhabditis* Genetics Center	Strain MT1306
Integrated line P*unc-17*::RFP	Josh Kaplan Lab (Babu et al., 2011)	*nuIs321*
Integrated line P*unc-25*::GFP	*Caenorhabditis* Genetics Center	*juIs76* CGC strain number CZ1200
Integrated line P*unc-129*::GFP::SNB-1	Josh Kaplan Lab (Sieburth et al., 2005)	*nuIs152*
Integrated line P*unc-*25::SNB-1::GFP	Josh Kaplan Lab (Hao et al., 2012)	*nuIs376*
Integrated line P*acr-2*::mCherry::RAB-3	Mike Francis Lab (Petrash et al., 2013)	*uIfs63* strain pPRB47
Integrated line P*unc-129*::SYD-2::YFP	Josh Kaplan Lab (Sieburth et al., 2005)	*nuIs159*
Integrated line P*unc-25*::SYD-2::GFP	*Caenorhabditis* Genetics Center (Yeh et al., 2005)	*hpIs3* CGC strain number ZM54
Integrated line P*myo-3*::UNC-29::GFP	Villu Maricq lab (Francis et al., 2005)	*akIs38*
Integrated line P*myo-3*::ACR-16::GFP	Josh Kaplan Lab (Babu et al., 2011)	*nuIs299*
Integrated line P*myo-3*::UNC-49::GFP	Josh Kaplan Lab (Babu et al., 2011)	*nuIs283*
Integrated line P*myo-3*::GCaMP3.35	*Caenorhabditis* Genetics Center (Schwarz et al., 2012)	*goels3* CGC strain number HBR4
Integrated line P*nab-1*::NAB-1::GFP	Mei Zhen Lab (Hung et al., 2007)	*hpIs66*
Integrated line P*unc-17*:: GFP-UtrCH	This study	*indIs001*
Integrated line P*unc-129*::LIN-44::mCherry	This study, array number *IndEx32* from (Pandey et al., 2017)	*indIs002*
Integrated line P*unc-17*::CWN-2::mCherry	This study	*indIs003*
Integrated line P*unc-17*::NLP-21::YFP	Derek Sieburth Lab (Staab et al., 2013)	*vjIs30*
Integrated line P*unc-129*::GSLN-1::YFP	Josh Kaplan Lab (Sieburth et al., 2005)	*nuIs169*
Plasmid BAB#0101 P*hic-1*::mCherry (injected into WT)	This study	Array *IndEx001*/strain BAB047
Plasmid BAB#0102 P*hic-1*::HIC-1::mCherry (injected into WT)	This study	Array *IndEx002*/strain BAB348
Plasmid BAB#0103 P*rab-3*::HIC-1 (injected into *hic-1*)	This study	Array *IndEx003*/strain BAB349
Plasmid BAB#0104 P*let-413*::HIC-1 (injected into *hic-1*)	This study	Array *IndEx004*/strain BAB350
Plasmid BAB#0105 P*myo-3*::HIC-1 (injected into *hic-1*)	This study	Array *IndEx005*/strain BAB351
Plasmid BAB#0106 *Punc-17::HIC-1* (injected into *hic-1*)	This study	Array *IndEx006*/strain BAB352
Plasmid BAB#0107 P*unc-25*::HIC-1 (injected into *hic-1*)	This study	Array *IndEx007*/strain BAB353
Plasmid BAB#0108 P*unc-17::HIC-1* ΔC(4aa) (injected into *hic-1*)	This study	Array *IndEx008*/strain BAB354
Plasmid BAB#0110 P*unc-17*::NAB-1::VN173 (injected into *uIfs63*)	This study	Array *IndEx009/* strain BAB355
Plasmid BAB#0111 P*unc-17*::HIC-1::VC155 (injected into *uIfs63*)	This study	Array *IndEx010/* strain BAB356
Plasmid BAB#0112 P*unc-17*::HIC-1ΔC(4aa)::VC155 (injected into *uIfs63*)	This study	Array *IndEx011/* strain BAB357
Plasmid BAB#0115 P*hic-1*::HIC-1ΔC(4aa)::mCherry (injected into *nuIs152*)	This study	Array *IndEx012/* strain BAB358
Plasmid BAB#0114 P*unc-25*:: GFP-UtrCH (injected into WT)	This study	Array *IndEx013/* strain BAB359
Plasmid BAB#0110 P*unc-17*::NAB-1::VN173 (injected into *nab-1*)	This study	Array *IndEx014/* strain BAB360
Plasmid BAB#0109 P*unc-17*::CWN-2::mCherry (injected into WT)	This study	Array *IndEx015/* strain BAB361
Plasmid BAB#0110, BAB#0111 P*unc-17::HIC-1*:: VC155;Punc17::NAB-1::VN173 (injected into *uIfs63*)	This study	Array *IndEx016/* strain BAB362
Plasmid BAB#0110, BAB#0112 P*unc-17*::HIC-1ΔC(4aa):: VC155; Punc17::NAB-1::VN173 (injected into *uIfs63*)	This study	Array *IndEx017/* strain BAB363
Plasmid BAB#0116 P*unc-17*:: HIC-1(ΔC(4aa)+NAB-1 (ABD)) (injected into BAB323)	This study	Array *IndEx018/* strain BAB364
Plasmid BAB#0116 P*unc-17*:: HIC-1(ΔC(4aa)+NAB-1 (ABD)) (injected into BAB330)	This study	Array *IndEx019/* strain BAB365
Plasmid BAB#0116 P*unc-17*:: HIC-1(ΔC(4aa)+NAB-1 (ABD)) (injected into BAB333)	This study	Array *IndEx020/* strain BAB366
Plasmid BAB#0108 P*unc-17::HIC-1* ΔC(4aa) (injected into BAB323)	This study	Array *IndEx021/* strain BAB367
Plasmid BAB#0108 P*unc-17*::HIC-1ΔC(4aa) (injected into BAB330)	This study	Array *IndEx022/* strain BAB368
Plasmid BAB#0108 P*unc-17*::HIC-1 ΔC(4aa) (injected into BAB333)	This study	Array *IndEx023/* strain BAB369
Plasmid *pCFJ68* P*unc-122*::GFP (injected into *indIs003*)	This study	Array *IndEx024/* strain BAB370
Plasmid *pCFJ68* P*unc-122*::GFP (injected into *indIs002*)	This study	Array *IndEx025/* strain BAB371
*hic-1(ok3475); nuIs321*	This study	BAB302
*hic-1(ok3475); juIs76*	This study	BAB303
*hic-1(ok3475); nuIs152*	This study	BAB304
*hic-1(ok3475); juIs376*	This study	BAB305
*hic-1(ok3475); uIfs63*	This study	BAB306
*hic-1(ok3475); nuIs159*	This study	BAB307
*hic-1(ok3475); hpIs3*	This paper	BAB308
*hic-1(ok3475); nuIs169*	This paper	BAB309
*hic-1(ok3475); nuIs169; IndEx006*	This study	BAB310
*hic-1(ok3475); akIs38*	This study	BAB311
*hic-1(ok3475); nuIs299*	This study	BAB312
*hic-1(ok3475); nuIs299; IndEx006*	This study	BAB313
*hic-1(ok3475); nuIs283*	This study	BAB314
*hic-1(ok3475); goels3*	This study	BAB315
*hic-1(ok3475); goels3; IndEx006*	This study	BAB316
*hic-1(ok3475); indIs001*	This study	BAB317
*hic-1(ok3475); indIs001; IndEx006*	This study	BAB318
*hic-1(ok3475); indIs002*	This study	BAB319
*hic-1(ok3475); indIs002; IndEx006*	This study	BAB320
*hic-1(ok3475); IndIs003*	This study	BAB321
*hic-1(ok3475); IndIs003; IndEx006*	This study	BAB322
*nab-1(ok943); hic-1(ok3475*)	This study	BAB323
*mig-14(mu71); hic-1(ok3475*)	This study	BAB324
*lin-17(sy277); hic-1(ok3475*)	This study	BAB325
*nab-1(ok943); IndEx014*	This study	BAB326
*nab-1(ok943); hpIs66*	This study	BAB327
*nab-1(ok943); IndIs001*	This study	BAB328
*nab-1(ok943); IndEx014; IndIs001*	This study	BAB329
*nab-1(ok943); hic-1(ok3475); IndIs001*	This study	BAB330
*nab-1(ok943); IndIs003*	This study	BAB331
*nab-1(ok943); IndEx014; IndIs003*	This study	BAB332
*nab-1(ok943); hic-1(ok3475); IndIs003*	This study	BAB333
*mig-14(mu71); IndIs003*	This study	BAB334
*mig-14(mu71); hic-1(ok3475); IndIs003*	This study	BAB335
*hic-1(ok3475); hpIs66*	This study	BAB336
*hpIs66; IndEx002*	This study	BAB337
*hic-1(ok3475); vjIs30*	This study	BAB348
*nab-1(ok943); nuIs299*	This study	BAB349
*nab-1(ok943); nuIs299; IndEx014*	This study	BAB350
*nab-1(ok943); hic-1(ok3475); nuIs299*	This study	BAB351
*hic-1(ok3475); acr-16(ok789*)	This study	BAB352
*hic-1(ok3475); hpIs66; IndEx006*	This study	BAB355
*hic-1(ok3475); IndEx024*	This study	BAB356
*hic-1(ok3475); IndEx006; IndEx024*	This study	BAB357
*mig-14(mu71); IndEx024*	This study	BAB358
*mig-14(mu71); hic-1(ok3475); IndEx024*	This study	BAB359
*hic-1(ok3475); IndEx025*	This study	BAB360
*hic-1(ok3475); IndEx006; IndEx025*	This study	BAB361
Oligonucleotides		
See Table S1 for primer sequences and information		N/A
Recombinant DNA		
*pPD49.26*	Addgene	Plasmid#1686 (Andrew Fire lab)
*pCFJ910*	Addgene	Plasmid#44481 (Erik Jorgensen lab)
*pCFJ68 (Exp5605_Punc-122::GFP::unc-54utr*)	Addgene	Plasmid#19325 (Erik Jorgensen lab)
*pCFJ90 (Pmyo-2::mCherry::unc-54utr*)	Addgene	Plasmid#19327 (Erik Jorgensen lab)
*pCFJ104 (Pmyo-3::mCherrry:::unc-54*)	Addgene	Plasmid#19328 (Erik Jorgensen lab)
*GFP-UtrCH* (reporter for F-actin)	Addgene	Plasmid#26737 (William Bement lab)
P*hic-1*::mCherry	This study	pBAB# 0101
P*hic-1*::HIC-1::mCherry	This study	pBAB# 0102
P*rab-3*::HIC-1	This study	pBAB# 0103
P*let-413*::HIC-1	This study	pBAB# 0104
P*myo-3*::HIC-1	This study	pBAB# 0105
P*unc-17*::HIC-1	This study	pBAB# 0106
P*unc-25*::HIC-1	This study	pBAB# 0107
P*unc-17*::HIC-1ΔC(4aa)	This study	pBAB# 0108
P*unc-17*::CWN-2::mCherry	This study	pBAB# 0109
P*unc-17*::NAB-1::Linker::VN173	This study	pBAB# 0110
P*unc-17*::HIC-1::Linker::VC155	This study	pBAB# 0111
P*unc-17*:: HIC-1ΔC(4aa)::Linker::VC155	This study	pBAB# 0112
P*unc-17*:: GFP-UtrCH	This study	pBAB# 0113
P*unc-25*:: GFP-UtrCH	This study	pBAB# 0114
P*hic-1* ::HIC-1ΔC(4aa)::mCherry	This study	pBAB# 0115
P*unc-17*:: HIC-1(ΔC(4aa)+NAB-1(ABD))	This study	pBAB# 0116
Software and Algorithms		
ImageJ	n/a	https://imagej.nih.gov/ij/
Prism7	Graphpad Prism	https://www.graphpad.com/
ApE	n/a	http://jorgensen.biology.utah.edu/wayned/ape/
Snapgene	Snapgene	http://www.snapgene.com/
Mind the graph	Mind the GRAPH	https://mindthegraph.com

### Contact for Reagent and Resource Sharing

Further information and requests for resources should be directed to and will be fulfilled by the Lead Contact, Dr. Kavita Babu (kavita.babu@babulab.org).

### Experimental Model and Subject Details

Organisms/Strains: *C.elegans* (WB Cat# N2_(ancestral), RRID:WB STRAIN:N2_(ancestral))

#### Strains

All strains were maintained on Nematode Growth Medium (NGM) plates seeded with OP50 *Escherichia coli* at 20°C under standard conditions (Brenner, 1974). The *C. elegans* Bristol strain N2 was used as the wild-type (WT) control. Mutant strains used were: *hic-1* (*ok3475*), *mig-14* (*ga62), lin-17* (*e1456*), *acr-16* (*ok789*), and *nab-1(ok943),* all available from CGC and outcrossed with N2 *C. elegans* at least 4X times. All the mutants and the primers for their genotyping are listed in the KRT table.

### Method Details

#### Behavioral assays

***Aldicarb assay***

This assay was performed as previously described (Mahoney et al., 2006). Briefly, the Aldicarb plates were prepared one day prior to the experiment, 100 mM stock of Aldicarb (Sigma-Aldrich, St. Louis, MO) was prepared in ethanol and incorporated in the NGM to make the final concentration of 1mM. At least 20 animals per genotype were picked at the late L4 stage for the assay. The number of paralyzed animals was counted at 10 minutes (min) intervals for up to 120 min. The assay was performed in triplicate with the experimenter being blind to the genotype of the *C. elegans*. Aldicarb assays were performed using multiple batches of Aldicarb. This gave rise to small discrepancies in the rate of paralysis of WT animals. However all the graphs were done with appropriate controls, with the experimenter being blind to the genotype of the animals, and showed a similar trend across the multiple batches of Aldicarb used.

***Sytox green uptake assay***

*C. elegans* were allowed to uptake sytox green dye at 22°C or 37°C for 1 hour (h). Images of the intestine of the *C. elegans* were captured using the 488nm filter and DIC after the sytox green treatment.

#### Microscopy and image analysis

***Neuronal and synaptic markers***

Imaging of neuronal and synaptic markers was done as previously described (Sieburth et al., 2005), by immobilizing *C. elegans* with 30mg/ml 2, 3-butanedione monoxamine (BDM) from Sigma on 2% agarose pads. A Zeiss AxioImager microscope with a 63x 1.4 NA Plan APOCHROMAT objective equipped with a Zeiss AxioCam MRm CCD camera controlled by Axiovision software (Zeiss Microimaging) was used for taking Z stacks of the *C.elegans*. Maximum intensity projections of the line-scans were taken for Quantitation. Images were then quantitated using ImageJ software. The Prism 7 software was used for the statistical analyses of the data.

The Colocalization studies and the GFP-UtrCH imaging experiments were performed on the Leica SP8 confocal microscope using an Argon laser at multiple gains ranging from 10% to 15%. All the parameters were kept constant for any one set of imaging experiments plotted on the same graph.

#### Fluorescent recovery after photobleaching (FRAP) Experiment

FRAP experiments were performed on late L4 stage *C. elegans* that were immobilized on 10% agarose pads. 0.2μl of 0.1 μm polystyrene beads (Polysciences) were added to the slides to restrict further the movement of the *C. elegans* as previously described (Babu et al., 2011). The SP8 confocal microscope (Leica Microsystems) with a 23% bleaching power of 488 nm Argon laser was used to bleach the ACR-16::GFP puncta. The images were captured before and after photobleaching for up to 20 min. The fluorescent intensities of neighboring puncta were also calculated and taken as a control to monitor the focal drift during the live imaging of the animals.

#### Coelomocyte uptake assay

The secretion of Wnt ligands from the cholinergic neurons was monitored using a technique similar to that used previously for Neuropeptide uptake assays (Sieburth et al., 2007) and as has been previously published for Wnts (Jensen et al., 2012). The fluorescence intensity of Wnts tagged with mCherry was measured in the endocytic compartment of coelomocytes posterior to the vulva of the WT and mutant *C. elegans*. Laterally oriented young adults were imaged in animals where the coelomocyte was not obscured by any other tissues. A maximum intensity projection was taken from image stacks of the coelomocyte, and the mean fluorescence within each vesicle in the coelomocyte was calculated using ImageJ software and plotted using Prism 7.

#### Latrunculin A (LAT-A) injections

The Lat-A injections for actin depolymerization were performed as described previously (Chia et al., 2012). L4 stage animals were immobilized by putting them on 2% agarose pads and adding halocarbon oil (Sigma). After immobilization, the *C. elegans* were injected with either 1mM of LAT-A (Sigma) in 25% vol/vol DMSO (Sigma) or 25% DMSO alone. The injections were done into the pseudocoelom of the *C. elegans* at a site slightly posterior to the vulva. The injected animals were then kept at 20°C for 3h to recover. After recovery, the animals were imaged as has been described above.

#### Calcium imaging

Calcium imaging in the *C.elegans* muscles was performed as described previously (Gong et al., 2016). Transgenic animals expressing *goeIs3* (P*myo-3*::GCaMP3.35::unc-54-3′utr,unc-119) were first immobilized on 2% agarose pads prepared in the M9 buffer. 0.2μl of polystyrene beads were further added to the agarose pads to restrict the movement of the animals. After immobilizing the *C. elegans*, the time-lapse movies were taken for the muscles at 63X on the Zeiss fluorescence upright microscope at 1 frame/second (s) speed for up to 40 s at 200ms exposure time. A complete cycle of calcium transients was taken for the analysis. Two parallel lines were drawn on the y axis at 10% and 90% from the baseline of the signal to the highest point of the peak (Petrou et al., 2017; Thrasivoulou et al., 2013) and represented in Figure S4B)). This makes four interception points on the waveform. The rise time was defined as the time from the initial point to the second point in seconds (*t1*s), the dwell time was calculated from the second point to the third point (*t2*s), and the fall time was reflected by the time from the third point to the fourth point (*t3*s). Data analysis was performed in the Prism 7 software after the images were analyzed using the ImageJ software.

#### Cloning and Constructs

Standard restriction digestion or PCR based cloning was performed for all the experiments in this study (Russell, 2001). The vectors used in this study are *pPD49.26*, *PCFJ910* and *PCFJ68* all obtained from Addgene. See also KRT table. A detailed description of all the constructs generated for the study and their respective cloning primers are indicated in the supplemental section Table S1.

#### BiFC Assay

To perform the BiFC assay, VN173 Fragment (N terminus) of split YFP (Kerppola, 2013) was cloned downstream to Neurabin cDNA, and VC155 Fragment (C terminus) of split YFP (Kerppola, 2013) was tagged to the C terminus of HIC-1 cDNA. For the control experiment, HIC-1ΔC(4aa)::VC155 was generated. All the constructs for BiFC experiments were cloned under the *unc-17* promoter to drive the expression in the cholinergic neurons. A brief summary of the sequences and the primers used for generating BiFC constructs are indicated in the supplemental section Table S2.

#### Generation of transgenics (extrachromosomal arrays and Integrated Lines)

*C.elegans* transformants were obtained as described previously (Mello and Fire, 1995). A 100 ng/ul concentration injection mix was prepared with 10-50 ng of the transgene DNA, 5-15 ng/ul of co-injection marker (*PCFJ90*) and rest of the concentration was made up with the pBluescript (PBS) plasmid.The extrachromosomal arrays with high-frequency transmission rate were integrated as described previously (Mariol et al., 2013). The list of integrated lines and their sources, list of arrays and the list of all the strains used in this work is listed in the Key Resource Table.

#### Quantitative PCR experiment

For real time PCR based gene expression analysis, Fresh RNA was isolated from a mixed staged population of *C. elegans* by initially treating the freeze-thawed *C. elegans* pellets with Trizol and then purifying the lysate with an RNeasy Mini Kit (QIAGEN). cDNA was synthesized from the isolated RNA with random hexamers using the Transcriptor High Fidelity cDNA Synthesis Kit (Roche) according to manufacturer’s protocols. SYBR Premix Ex TaqII master mix (Clontech) was used to set up the real time PCR reaction in triplicate. The normalized expression of genes in different mutants relative to WT controls was calculated using the 2^−ΔΔCt^ method.

#### Electrophysiology Experiments

*C.elegans* dissection, neuromuscular junction exposure and Whole-cell voltage-clamp recordings were performed on body-wall muscles as previously described (Hu et al., 2012; Richmond and Jorgensen, 1999). We recorded miniature excitatory postsynaptic currents by perfusing *C. elegans* in an extracellular recording solution made up of 127mM NaCl, 5mM KCl, 26mM NAHCO_3_, 1.25mM NAH_2_PO_4_, 20mM glucose, 1mM CaCl_2_, and 4mM MgCl_2_, bubbled with 5%CO_2_, 95%O_2_ at 22°C. The holding potential was set at −60mV, which is the reversal potential for the GABA_A_ receptors. In this way, only mEPSCs were recorded (Hu et al., 2013). The intracellular recording solution consists of 105mM CsCH_3_SO_3_, 10mM CsCl, 15mM CsF, 4mM MgCl_2_, 5mM EGTA, 0.25mM CaCl_2_, 10mM HEPES, and 4mM Na_2_ATP at pH7.2 adjusted by adding CsOH. Stimulus-evoked EPSCs were stimulated by placing a borosilicate pipette (5μm in open size) near the ventral nerve cord (one muscle distance from the recording pipette) and applying a 0.4 ms, 85μA square pulse (WPI).

### Quantification and Statistical Analysis

All statistical analysis was performed using GraphPad Prism V7. All experimental data are shown as mean ± SEM unless otherwise stated. Statistical comparisons were done using the Student’s t test, two-way ANOVA, one-way ANOVA with Bonferroni’s multiple comparison post test or Kolmogorov–Smirnov test (KS-test). A level of p < 0.05 was considered significant. Multiple sequence Alignment among claudin homologs or HIC-1 with other tetraspan proteins were analyzed using the Clustal Omega program (Sievers et al., 2011).

## Supplementary Material

Supplemental Information includes seven figures and two tables and can be found with this article online at https://doi.org/10.1016/j.celrep.2018.10.053.

SI file

## Highlights

- HIC-1 is expressed in cholinergic synapses and required for normal NMJ function
- Loss of *hic-1* causes defects in the actin cytoskeleton and in Wnt secretion
- HIC-1 functions through the actin-binding protein Neurabin at cholinergic synapses

## References

[R1] Abbas L (2003). Synapse formation: let’s stick together. Curr Biol.

[R2] Alfonso A, Grundahl K, Duerr JS, Han HP, Rand JB (1993). The Caenorhabditis elegans unc-17 gene: a putative vesicular acetylcholine transporter. Science.

[R3] Babu K, Hu Z, Chien SC, Garriga G, Kaplan JM (2011). The immunoglobulin super family protein RIG-3 prevents synaptic potentiation and regulates Wnt signaling. Neuron.

[R4] Barik A, Zhang B, Sohal GS, Xiong WC, Mei L (2014). Crosstalk between Agrin and Wnt signaling pathways in development of vertebrate neuromuscular junction. Dev Neurobiol.

[R5] Bernstein BW, Bamburg JR (1989). Cycling of actin assembly in synaptosomes and neurotransmitter release. Neuron.

[R6] Brenner S (1974). The genetics of Caenorhabditis elegans. Genetics.

[R7] Brumwell CL, Johnson JL, Jacob MH (2002). Extrasynaptic α 7-nicotinic acetylcholine receptor expression in developing neurons is regulated by inputs, targets, and activity. J Neurosci.

[R8] Burkel BM, von Dassow G, Bement WM (2007). Versatile fluorescent probes for actin filaments based on the actin-binding domain of utrophin. Cell Motil Cytoskeleton.

[R9] Cadigan KM, Nusse R (1997). Wnt signaling: a common theme in animal development. Genes Dev.

[R10] Chatterjee I, Richmond A, Putiri E, Shakes DC, Singson A (2005). The Caenorhabditis elegans spe-38 gene encodes a novel four-pass integral membrane protein required for sperm function at fertilization. Development.

[R11] Chia PH, Patel MR, Shen K (2012). NAB-1 instructs synapse assembly by linking adhesion molecules and F-actin to active zone proteins. Nat Neurosci.

[R12] Coué M, Brenner SL, Spector I, Korn ED (1987). Inhibition of actin polymerization by latrunculin A. FEBS Lett.

[R13] Dorsch S, Klotz KN, Engelhardt S, Lohse MJ, Bünemann M (2009). Analysis of receptor oligomerization by FRAP microscopy. Nat Methods.

[R14] Eisenmann DM, Kim SK (2000). Protruding vulva mutants identify novel loci and Wnt signaling factors that function during Caenorhabditis elegans vulva development. Genetics.

[R15] Fares H, Greenwald I (2001). Genetic analysis of endocytosis in Caenorhabditis elegans: coelomocyte uptake defective mutants. Genetics.

[R16] Francis MM, Evans SP, Jensen M, Madsen DM, Mancuso J, Norman KR, Maricq AV (2005). The Ror receptor tyrosine kinase CAM-1 is required for ACR-16-mediated synaptic transmission at the C. elegans neuromuscular junction. Neuron.

[R17] Frøkjaer-Jensen C, Davis MW, Hopkins CE, Newman BJ, Thummel JM, Olesen S-P, Grunnet M, Jorgensen EM (2008). Single-copy insertion of transgenes in Caenorhabditis elegans. Nat Genet.

[R18] Gill MS, Olsen A, Sampayo JN, Lithgow GJ (2003). An automated high-throughput assay for survival of the nematode Caenorhabditis elegans. Free Radic Biol Med.

[R19] Gonçalves A, Ambrósio AF, Fernandes R (2013). Regulation of claudins in blood-tissue barriers under physiological and pathological states. Tissue Barriers.

[R20] Gong J, Yuan Y, Ward A, Kang L, Zhang B, Wu Z, Peng J, Feng Z, Liu J, Xu XZ (2016). The C. elegans taste receptor homolog LITE-1 is a photoreceptor. Cell.

[R21] Günzel D, Yu AS (2013). Claudins and the modulation of tight junction permeability. Physiol Rev.

[R22] Hagen SJ (2017). Non-canonical functions of claudin proteins: beyond the regulation of cell-cell adhesions. Tissue Barriers.

[R23] Hammond C, Denzin LK, Pan M, Griffith JM, Geuze HJ, Cresswell P (1998). The tetraspan protein CD82 is a resident of MHC class II compartments where it associates with HLA-DR, -DM, and -DO molecules. J Immunol.

[R24] Hao Y, Hu Z, Sieburth D, Kaplan JM (2012). RIC-7 promotes neuropeptide secretion. PLoS Genet.

[R25] Hardin J, King RS (2008). The long and the short of Wnt signaling in C. elegans. Curr Opin Genet Dev.

[R26] Henriquez JP, Webb A, Bence M, Bildsoe H, Sahores M, Hughes SM, Salinas PC (2008). Wnt signaling promotes AChR aggregation at the neuromuscular synapse in collaboration with agrin. Proc Natl Acad Sci USA.

[R27] Herr P, Hausmann G, Basler K (2012). WNT secretion and signalling in human disease. Trends Mol Med.

[R28] Hu Z, Hom S, Kudze T, Tong XJ, Choi S, Aramuni G, Zhang W, Kaplan JM (2012). Neurexin and neuroligin mediate retrograde synaptic inhibition in C. elegans. Science.

[R29] Hu Z, Tong XJ, Kaplan JM (2013). UNC-13L, UNC-13S, and Tomosyn form a protein code for fast and slow neurotransmitter release in Caenorhabditis elegans. eLife.

[R30] Hua VB, Chang AB, Tchieu JH, Kumar NM, Nielsen PA, Saier MH (2003). Sequence and phylogenetic analyses of 4 TMS junctional proteins of animals: connexins, innexins, claudins and occludins. J Membr Biol.

[R31] Hung W, Hwang C, Po MD, Zhen M (2007). Neuronal polarity is regulated by a direct interaction between a scaffolding protein, Neurabin, and a presynaptic SAD-1 kinase in Caenorhabditis elegans. Development.

[R32] Hussaini SM, Choi CI, Cho CH, Kim HJ, Jun H, Jang MH (2014). Wnt signaling in neuropsychiatric disorders: ties with adult hippocampal neurogenesis and behavior. Neurosci Biobehav Rev.

[R33] Jacob TC, Kaplan JM (2003). The EGL-21 carboxypeptidase E facilitates acetylcholine release at Caenorhabditis elegans neuromuscular junctions. J Neurosci.

[R34] Jensen M, Hoerndli FJ, Brockie PJ, Wang R, Johnson E, Maxfield D, Francis MM, Madsen DM, Maricq AV (2012). Wnt signaling regulates acetylcholine receptor translocation and synaptic plasticity in the adult nervous system. Cell.

[R35] Kahn M (2014). Can we safely target the WNT pathway?. Nat Rev Drug Discov.

[R36] Kamimura K, Ueno K, Nakagawa J, Hamada R, Saitoe M, Maeda N (2013). Perlecan regulates bidirectional Wnt signaling at the Drosophila neuromuscular junction. J Cell Biol.

[R37] Kerppola TK (2013). Bimolecular fluorescence complementation (BiFC) analysis of protein interactions in live cells. Cold Spring Harb Protoc.

[R38] Klassen MP, Shen K (2007). Wnt signaling positions neuromuscular connectivity by inhibiting synapse formation in C. elegans. Cell.

[R39] Kojima T, Yamaguchi H, Ito T, Kyuno D, Kono T, Konno T, Sawada N (2013). Tight junctions in human pancreatic duct epithelial cells. Tissue Barriers.

[R40] Koles K, Budnik V (2012a). Exosomes go with the Wnt. Cell Logist.

[R41] Koles K, Budnik V (2012b). Wnt signaling in neuromuscular junction development. Cold Spring Harb Perspect Biol.

[R42] Krause G, Winkler L, Mueller SL, Haseloff RF, Piontek J, Blasig IE (2008a). Structure and function of claudins. Biochimica et Biophysica Acta (BBA) - Biomembranes.

[R43] Krause G, Winkler L, Mueller SL, Haseloff RF, Piontek J, Blasig IE (2008b). Structure and function of claudins. Biochim Biophys Acta.

[R44] Liégeois S, Benedetto A, Michaux G, Belliard G, Labouesse M (2007). Genes required for osmoregulation and apical secretion in Caenorhabditis elegans. Genetics.

[R45] Logan CY, Nusse R (2004). The Wnt signaling pathway in development and disease. Annu Rev Cell Dev Biol.

[R46] Maguschak KA, Ressler KJ (2012). A role for WNT/β-catenin signaling in the neural mechanisms of behavior. J Neuroimmune Pharmacol.

[R47] Mahoney TR, Luo S, Nonet ML (2006). Analysis of synaptic transmission in Caenorhabditis elegans using an aldicarb-sensitivity assay. Nat Protoc.

[R48] Mariol MC, Walter L, Bellemin S, Gieseler K (2013). A rapid protocol for integrating extrachromosomal arrays with high transmission rate into the C. elegans genome. J Vis Exp.

[R49] McIntire SL, Jorgensen E, Horvitz HR (1993a). Genes required for GABA function in Caenorhabditis elegans. Nature.

[R50] McIntire SL, Jorgensen E, Kaplan J, Horvitz HR (1993b). The GABAergic nervous system of Caenorhabditis elegans. Nature.

[R51] Mello C, Fire A (1995). DNA transformation. Methods Cell Biol.

[R52] Messéant J, Ezan J, Delers P, Glebov K, Marchiol C, Lager F, Renault G, Tissir F, Montcouquiol M Sans N (2017). Wnt proteins contribute to neuromuscular junction formation through distinct signaling pathways. Development.

[R53] Morales M, Colicos MA, Goda Y (2000). Actin-dependent regulation of neurotransmitter release at central synapses. Neuron.

[R54] Nakanishi H, Obaishi H, Satoh A, Wada M, Mandai K, Satoh K, Nishioka H, Matsuura Y, Mizoguchi A, Takai Y (1997). Neurabin: a novel neural tissue-specific actin filament-binding protein involved in neurite formation. J Cell Biol.

[R55] Nomme J, Antanasijevic A, Caffrey M, Van Itallie CM, Anderson JM, Fanning AS, Lavie A (2015). Structural basis of a key factor regulating the affinity between the zonula occludens first PDZ domain and claudins. J Biol Chem.

[R56] Pandey P, Bhardwaj A, Babu K (2017). Regulation of WNT signaling at the neuromuscular junction by the immunoglobulin superfamily protein RIG-3 in *Caenorhabditis elegans*. Genetics.

[R57] Petrash HA, Philbrook A, Haburcak M, Barbagallo B, Francis MM (2013). ACR-12 ionotropic acetylcholine receptor complexes regulate inhibitory motor neuron activity in Caenorhabditis elegans. J Neurosci.

[R58] Petrou T, Olsen HL, Thrasivoulou C, Masters JR, Ashmore JF, Ahmed A (2017). Intracellular calcium mobilization in response to ion channel regulators via a calcium-induced calcium release mechanism. J Pharmacol Exp Ther.

[R59] Reits EA, Neefjes JJ (2001). From fixed to FRAP: measuring protein mobility and activity in living cells. Nat Cell Biol.

[R60] Richmond JE, Jorgensen EM (1999). One GABA and two acetylcholine receptors function at the C. elegans neuromuscular junction. Nat Neurosci.

[R61] Russell JSaD (2001). Molecular Cloning: A Laboratory Manual.

[R62] Schwarz J, Spies JP, Bringmann H (2012). Reduced muscle contraction and a relaxed posture during sleep-like Lethargus. Worm.

[R63] Sharma P, Li L, Liu H, Tikiyani V, Hu Z, Babu K (2018). The claudin-like protein HPO-30 is required to maintain LAChRs at the *C. elegans* neuromuscular junction. J Neurosci.

[R64] Sieburth D, Ch’ng Q, Dybbs M, Tavazoie M, Kennedy S, Wang D, Dupuy D, Rual JF, Hill DE, Vidal M (2005). Systematic analysis of genes required for synapse structure and function. Nature.

[R65] Sieburth D, Madison JM, Kaplan JM (2007). PKC-1 regulates secretion of neuropeptides. Nat Neurosci.

[R66] Sievers F, Wilm A, Dineen D, Gibson TJ, Karplus K, Li W, Lopez R, McWilliam H, Remmert M, Söding J (2011). Fast, scalable generation of high-quality protein multiple sequence alignments using Clustal Omega. Mol Syst Biol.

[R67] Simske JS (2013). Claudins reign: the claudin/EMP/PMP22/γ channel protein family in C. elegans. Tissue Barriers.

[R68] Staab TA, Griffen TC, Corcoran C, Evgrafov O, Knowles JA, Sieburth D (2013). The conserved SKN-1/Nrf2 stress response pathway regulates synaptic function in Caenorhabditis elegans. PLoS Genet.

[R69] Suzuki H, Nishizawa T, Tani K, Yamazaki Y, Tamura A, Ishitani R, Dohmae N, Tsukita S, Nureki O, Fujiyoshi Y (2014). Crystal structure of a claudin provides insight into the architecture of tight junctions. Science.

[R70] Tallafuss A, Constable JR, Washbourne P (2010). Organization of central synapses by adhesion molecules. Eur J Neurosci.

[R71] Thorpe CJ, Schlesinger A, Carter JC, Bowerman B (1997). Wnt signaling polarizes an early C. elegans blastomere to distinguish endoderm from mesoderm. Cell.

[R72] Thrasivoulou C, Millar M, Ahmed A (2013). Activation of intracellular calcium by multiple Wnt ligands and translocation of β-catenin into the nucleus: a convergent model of Wnt/Ca2+ and Wnt/β-catenin pathways. J Biol Chem.

[R73] Tsukita S, Furuse M (2000). The structure and function of claudins, cell adhesion molecules at tight junctions. Ann N Y Acad Sci.

[R74] Umeda K, Ikenouchi J, Katahira-Tayama S, Furuse K, Sasaki H, Nakayama M, Matsui T, Tsukita S, Furuse M, Tsukita S (2006). ZO-1 and ZO-2 independently determine where claudins are polymerized in tight-junction strand formation. Cell.

[R75] Veltri A, Lang C, Lien WH (2017). Concise review: Wnt signaling pathways in skin development and epidermal stem cells. Stem Cells.

[R76] Wang W, Xu L, Liu P, Jairam K, Yin Y, Chen K, Sprengers D, Peppelenbosch MP, Pan Q, Smits R (2016a). Blocking Wnt secretion reduces growth of hepatocellular carcinoma cell lines mostly independent of β-catenin signaling. Neoplasia.

[R77] Wang Z, Li B, Zhou L, Yu S, Su Z, Song J, Sun Q, Sha O, Wang X, Jiang W (2016b). Prodigiosin inhibits Wnt/β-catenin signaling and exerts anticancer activity in breast cancer cells. Proc Natl Acad Sci U S A.

[R78] Yamagata M, Sanes JR, Weiner JA (2003). Synaptic adhesion molecules. Curr Opin Cell Biol.

[R79] Yeh E, Kawano T, Weimer RM, Bessereau JL, Zhen M (2005). Identification of genes involved in synaptogenesis using a fluorescent active zone marker in Caenorhabditis elegans. J Neurosci.

[R80] Zhang W, Benson DL (2002). Developmentally regulated changes in cellular compartmentation and synaptic distribution of actin in hippocampal neurons. J Neurosci Res.

[R81] Zhang B, Liang C, Bates R, Yin Y, Xiong WC, Mei L (2012). Wnt proteins regulate acetylcholine receptor clustering in muscle cells. Mol Brain.

[R82] Zou W, Dong X, Broederdorf TR, Shen A, Kramer DA, Shi R, Liang X, Miller DM, Xiang YK, Yasuda R (2018). A dendritic guidance receptor complex brings together distinct actin regulators to drive efficient F-actin assembly and branching. Dev Cell.

