# Supplementary material for: Wnt Secretion Is Regulated by the Tetraspan Protein HIC-1 through Its Interaction with Neurabin/NAB-1
Source: Cell Rep. Author manuscript; Available in PMC 2018 Nov 28. (PMC6258899; doi:10.1016/j.celrep.2018.10.053)
Supplement: SI file [file NIHMS80601-supplement-SI_file.pdf]

A

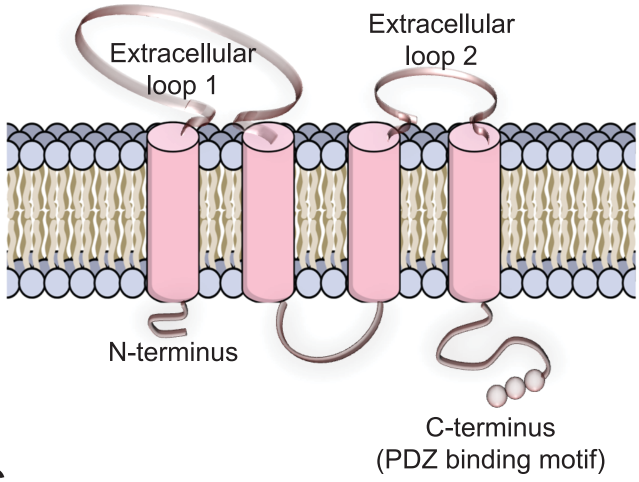

B

Alignment of the PDZ binding domain

```
Mclaudin-5    --KNYV-
Hclaudin-5    --KNYV-
Hclaudin-3    --KDYV-
clc-5         ---FYSY
clc-4         QHGV---
hic-1         --SEYR-
clc-1         EHCC---
nsy-4         ETCV---
clc-2         -TTVF--
clc-3         VTEV---
```

C

Alignment of the Extracellular Loop 1

```
clc-3        MHRG-VLRQCITTRQYG-SCNFR-----LSSMFKQLR
clc-1        EWNGLMPWSCIGHSQGT-QCQDWWANLPG--------
clc-4        RLTGILPFLCEDGA--V-GCAGFWKE-----------
Hclaudin-3   IWEG-LWMNCVVQSTGQMQCKVYDSLLALPQDLQAAR
Mclaudin-5   TWKG-LWMSCVVQSTGHMQCKVYESVLALSAEVQAAR
Hclaudin-5   TWKG-LWMSCVVQSTGHMQCKVYDSVLALSTEVQAAR
hic-1        QRSA-GWLQCVVVCQLMAFSFE---------------
clc-2        VQSG-LWLSCQTRPNGMYSCTYTFSHDDFNTYF----
clc-5        VQSG-LWLYCPGQA----QCWYIFSDSLINYY-----
hpo-30       HHHG-LWWDCIVHHETLIPLHEDQAELR---------
nsy-4        HEHG-LWLDCTRHSRDGNHILQRYATV----------
```

D

CLUSTAL O(1.2.4) multiple sequence alignment

```
CD82    39   ------------------------------------------------------MGSACI KVTKYFLFLFNLI FFILGAVI LGFGVWILADKSSFISVLQTSSSSLRMGAYVF IGVGAVT------MLMGFLGCIGAVNE
RDY-2 114    -----------------GQNVYGQVALI CI TLAFGLITVGVSCVTLATGSILTYPQILISG--------LSMVAF IVT----GG-VET-WYATGYDHMEFF----IQAVGNGVFSGCAGIPGCQIQFVV
HIC-1   26   ----------------------------------MHHHHHHAPFA--------------------- IAGLL---------------------------I I VT ILTGVGTFTNY-WGVSGNLHMGIYQWGQAGANRSFQRSAGWLQCVVVCQL
SPE38   49   NDANYTVFCCSHVVLAFA---------------------IFMILDFTLTLF-S---------FGLELF LI FFHDTFDQF-WAIFHGISLVTS-------------------------I SAVLA I CDNPVAI

CD82         -----VRCLLGLYFAFLLL I L I------------------------------------------------------------AQVTAGALFYFNMGKLKQEMGGIVTELIRDYNSSREDSLQDAWDYVQA-----------------QVKC
RDY-2        KGWAVAAAFYFLGALLYVVDMSL I FISRENEPAPP-----------------------------------------------------------------------------------------------------------
HIC-1        MAFSFEL---------LFCLLV I-----PA IVFRRMMPVHAACTLLSL I I FILLL I SIIVFAANIGSFYYNALISLKL-----------------------------------------GWSWGI TLAATILSFLLLLVSGSA
SPE38        IVVYGCRV--------MLILGFVTTRF I ISMRDFTSKEEPTLSELLNL----FQIVIV-------------------------------I--------------------------------------- MYT I GI FLV IR-----------LFTYSA

CD82         CGWVSFYNWTDNAELMNRPEVTYPCSCEVKGEEDNSLSVRKGFCEAPGNRTQSGNHPEDWPVYQEGCMEKVQAWLQENLGIILGVGVGVAIIELLGMVLSICLCRH   255
RDY-2        VVFRGNR-------------------------------------------------------------TI RSSF---------------------------------------------------------------------------------------------------   202
HIC-1        TGYGAYSEYR-----------------------------------------------------------------------------------------------------------------------------------------------------------------------   167
SPE38        GRYRGYYDVDDDL-------------------------------------------------KDEIRKKFLEYEADLDEKSEKKD---------------------------------------------------------------------------------------   179
```

E

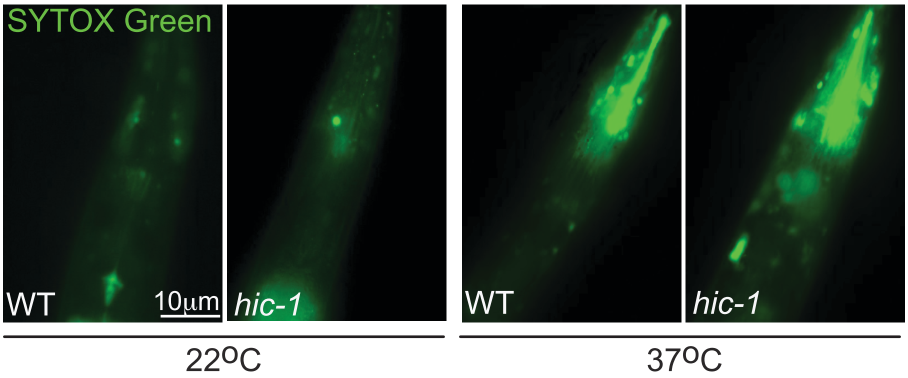

Figure S1. Putative structure of HIC-1. Related to Figure 1.
A. An illustration showing the structure of a typical claudin.
B. Multialignment of the C-terminus PDZ binding motif (bm) of different claudins indicating the conservation of the critical Tyrosine residue in HIC-1.
C. Multialignment of the extracellular loop 1 of different claudins shows the absence of the critical "GLW" residues in HIC-1 (This shows instead the "GWL" residues). The "C" amino acid is however conserved in HIC-1.
D. Multialignment of HIC-1 with tetraspan proteins RYD-2, SPE-38, and CD82. HIC-1 does not show the presence of conserved motifs with these proteins.
E. Representative fluorescent images of the intestine after sytox green dye uptake at 22oC and 37oC in wild type (WT) and *hic-1* mutant animals. Five animals were tested for each genotype and temperature.

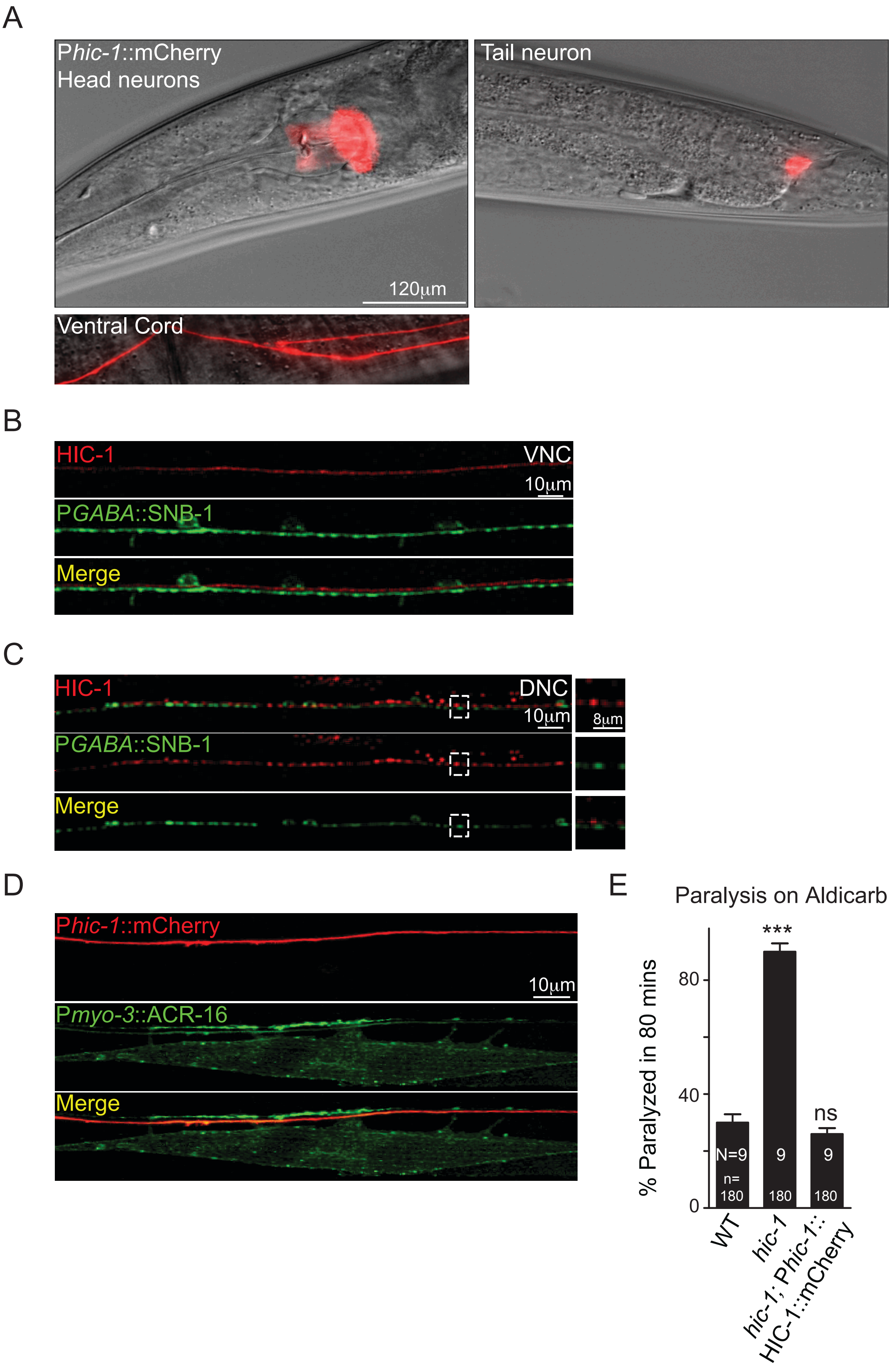
A
Phic-1::mCherry
Head neurons
120µm
Tail neuron
Ventral Cord
B
HIC-1
VNC
10µm
PGABA::SNB-1
Merge
C
HIC-1
DNC
10µm
8µm
PGABA::SNB-1
Merge
D
Phic-1::mCherry
10µm
Pmyo-3::ACR-16
Merge
E
Paralysis on Aldicarb
% Paralyzed in 80 mins
0
40
80
***
ns
N=9
9
9
n=
180
180
180
WT
hic-1
hic-1; Phic-1::
HIC-1::mCherry

Figure S2. Localization of HIC-1. Related to Figure 1.

A. Expression of P*hic-1*::mCherry in *C.elegans*. The representative images were obtained with DIC and a 587nm excitation filter show P*hic-1*::mCherry expression in head neurons and tail neurons. mCherry expression was also seen along the ventral nerve cord (VNC).

B. The punctate expression of the HIC-1::mCherry translational reporter does not appear to overlap with the presynaptic expression of SNB-1::GFP expressed along the VNC of GABA neurons.

C. No obvious co-localization was seen along the Dorsal Nerve Cord (DNC) between the HIC-1::mCherry translational reporter and SNB-1::GFP expressed in GABA neurons. The boxes in each image indicate the regions shown on the right of the image. More than10 animals were imaged for this experiment.

D. HIC-1 does not show expression in the body-wall muscles. A representative image of the body-wall muscle of an animal expressing P*myo-3*::ACR-16::GFP and P*hic-1*::mCherry. n>10.

E. Aldicarb assay indicating paralysis at 80 minutes (m) of the following genotypes; WT, *hic-1* and *hic-1*; P*hic-1*::HIC-1::mCherry. The numbers at the base of all Aldicarb bar graphs are denoted as N= number of trials performed and n= total number of animal used in each experiment (~20 animals/trial) for each genotype. The Data are represented as mean ± SEM and p values calculated using one-way ANOVA and Bonferroni's Multiple Comparison Test. ***p<0.001 and "ns" indicates not significant in all figures.

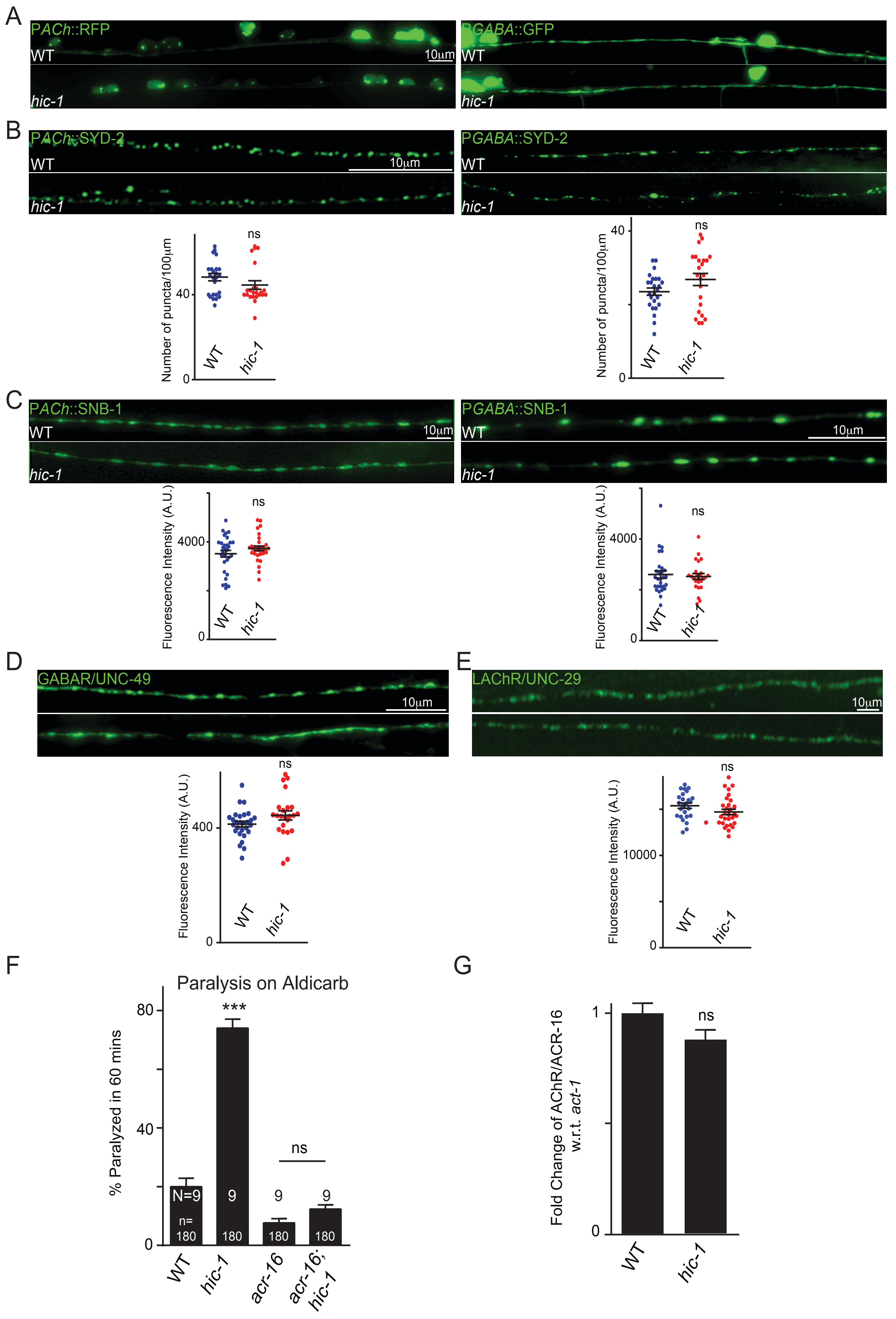
A
PACh::RFP
WT
hic-1
10µm
PGABA::GFP
WT
hic-1
B
PACh::SYD-2
WT
hic-1
10µm
PGABA::SYD-2
WT
hic-1
Number of puncta/100µm
0
40
ns
WT
hic-1
Number of puncta/100µm
0
40
ns
WT
hic-1
C
PACh::SNB-1
WT
hic-1
10µm
PGABA::SNB-1
WT
hic-1
10µm
Fluorescence Intensity (A.U.)
0
4000
ns
WT
hic-1
Fluorescence Intensity (A.U.)
0
4000
ns
WT
hic-1
D
GABAR/UNC-49
10µm
Fluorescence Intensity (A.U.)
0
400
ns
WT
hic-1
E
LAChR/UNC-29
10µm
Fluorescence Intensity (A.U.)
0
10000
ns
WT
hic-1
F
Paralysis on Aldicarb
% Paralyzed in 60 mins
0
40
80
***
ns
N=9
9
9
9
n=
180
180
180
180
WT
hic-1
acr-16
acr-16; hic-1
G
Fold Change of AChR/ACR-16 w.r.t. act-1
0
1
ns
WT
hic-1

Figure S3. Neuronal and synaptic development are largely unaffected in *hic-1* mutants. Related to Figure 1.

A. Representative fluorescent images of cholinergic (P*ACh*::RFP) and GABAergic (P*GABA*::GFP) neurons along the VNC in wild type (WT) and *hic-1* mutants. Number of animals imaged with P*ACh*::RFP were WT (n=25) and *hic-1* (n=20). Number of *C. elegans* imaged with P*GABA*::GFP were WT (n=23) and *hic-1* (n=18).

B. Representative fluorescent images and quantitation of the density of fluorescent puncta of α-LIPRIN/SYD-2 expression in cholinergic and GABAergic synapses along the DNC in WT and *hic-1* mutants. The data are shown as mean ± SEM and the p value calculated using two-tailed unpaired student's t-test. For cholinergic synapses animals imaged were n=25 (WT) and n=23 (*hic-1*) and *C. elegans* imaged for GABAergic synapses were n=25 (WT) and n=23 (*hic-1*).

C. Representative images and dot plots of the fluorescence intensity of presynaptic SNB-1::GFP expressed in cholinergic and GABA synapses in WT and *hic-1* mutant animals. The number of animals imaged per genotype using the cholinergic SNB-1 marker were n=30 (WT) and n= 32 (*hic-1*). The number of animals imaged per genotype using the GABAergic SNB-1 marker were n=28 (WT) and n=25 (*hic-1*). Statistics used was the two-tailed unpaired student's t-test.

D. Representative fluorescent images and quantitation of the fluorescent intensity along the DNC of GABAR::GFP expressed in the body-wall muscles in WT and *hic-1* mutant *C. elegans*. The data are shown as mean ± SEM and the p value calculated using two-tailed unpaired student's t-test. Animals imaged were n=28 (WT) and n=23 (*hic-1*).

E. Representative images and quantitation of fluorescence intensities along the VNC of the animals expressing the LAChR/UNC-29::GFP transgene. Animals imaged were n=25 (WT) and n=30 (hic-1). The p value was calculated using two-tailed unpaired student's t-test.

F. Bar graph of Aldicarb assays of *AChR/acr-16; hic-1* animals. Mutants in *AChR/acr-16* completely suppress the hypersensitivity to Aldicarb seen in *hic-1* mutants.

G. Bar graph of a quantitative real time PCR experiment showing the fold change in the *AChR/ACR-16* cDNA levels in WT and *hic-1* mutants with respect to the *act-1* gene as an internal control. The data are shown as mean ± SEM and the p value calculated using two-tailed unpaired student's t-test.

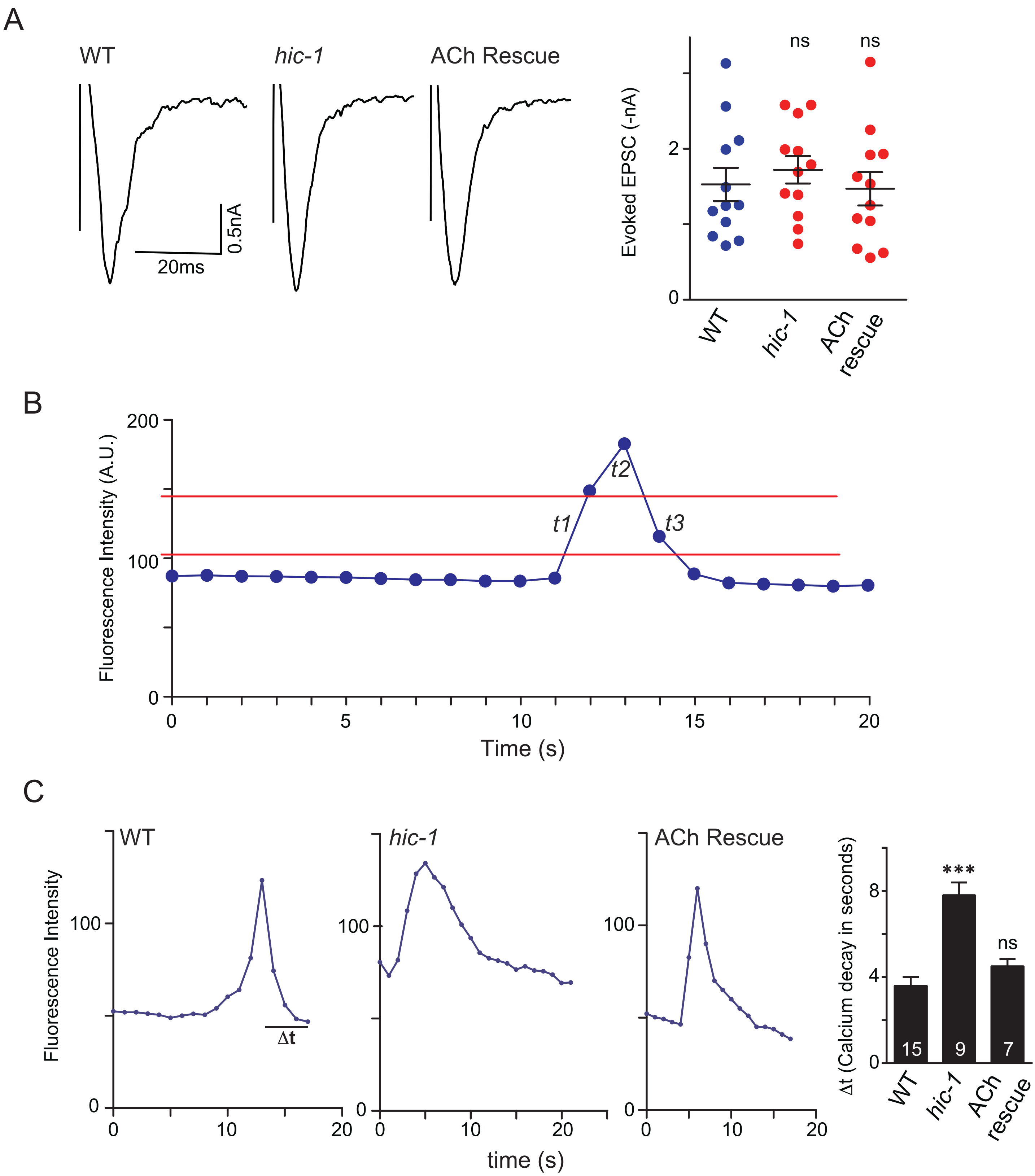

Figure S4. Mutants in *hic-1* show aberrant muscle responsiveness. Related to Figure 2.
A. Representative traces and summary data for evoked EPSC peak amplitude in WT and *hic-1* mutant *C. elegans*, n=12 (WT), n=12 (*hic-1*) and n=12 (*hic-1*; P*ACh*::HIC-1). The Data are represented as mean ± SEM and p values calculated using one-way ANOVA and Bonferroni's Multiple Comparison Test. In the dot plot, "ns" indicates not significant in comparison to WT values.
B. Representative trace of a calcium transient in WT *C. elegans* and the representation of the different time constants used, as has been previously published (Petrou et al., 2017).
C. Representative calcium transients in WT, *hic-1* and *hic-1*; P*ACh*::HIC-1 animals. The calcium decay time (Δt) refers to change in time between the maximum intensity peak to the minimum fall of the intensity. The bar graph shows the average calcium decay time in WT, *hic-1* and *hic-1*; P*ACh*::HIC-1 animals. The numbers at the base of the graph indicates the number of animals analyzed. The Data are represented as mean ± SEM and p values calculated using one-way ANOVA and Bonferroni's Multiple Comparison Test. In the bar graph, "***" indicates p<0.001 and "ns" indicates not significant in comparison to WT values.

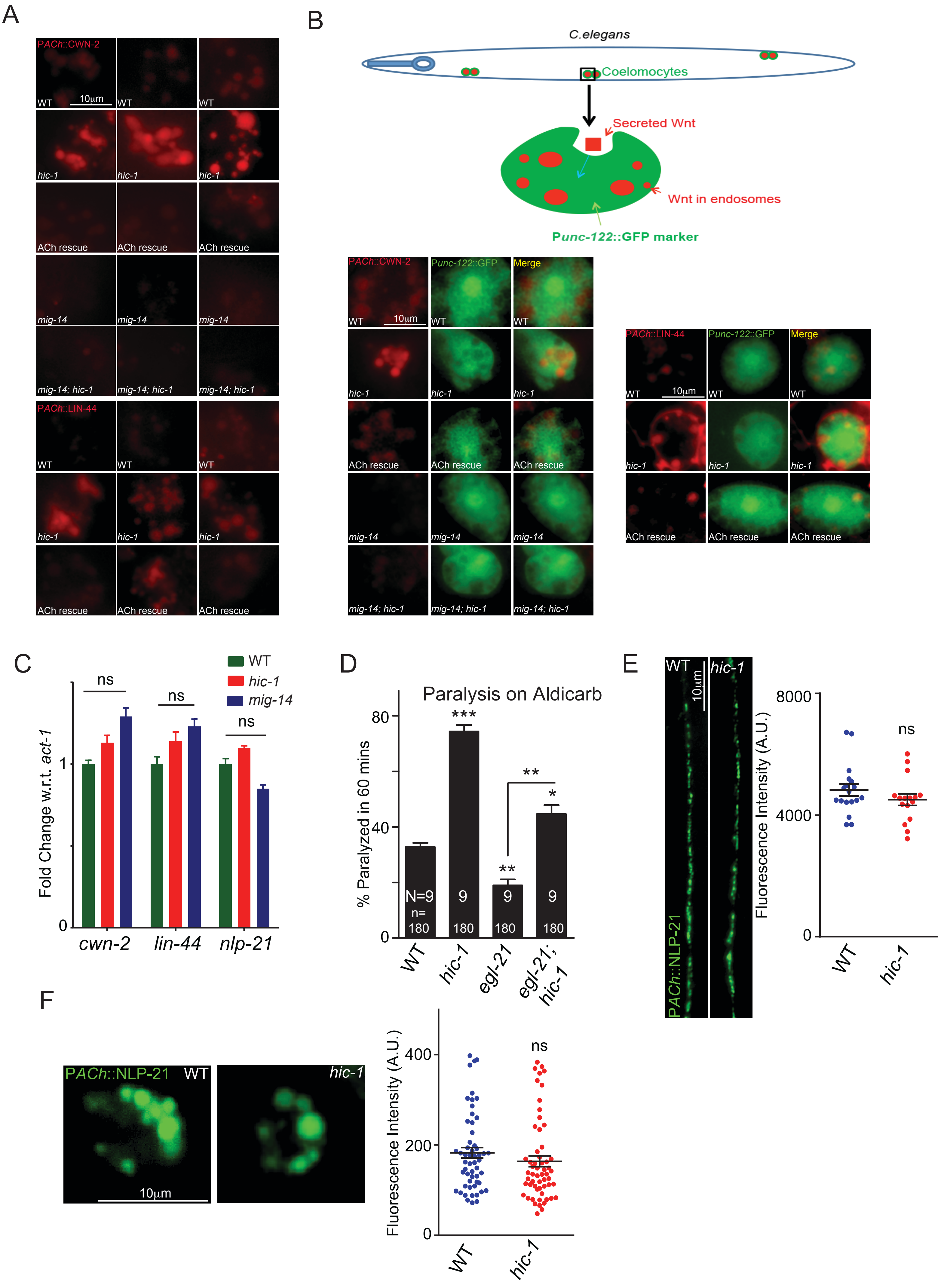

A
PACh::CWN-2
WT
10µm
hic-1
ACh rescue
mig-14
mig-14; hic-1
PACh::LIN-44
B
C.elegans
Coelomocytes
Secreted Wnt
Wnt in endosomes
Punc-122::GFP marker
PACh::CWN-2
Punc-122::GFP
Merge
PACh::LIN-44
C
WT
hic-1
mig-14
ns
Fold Change w.r.t. act-1
cwn-2
lin-44
nlp-21
D
Paralysis on Aldicarb
% Paralyzed in 60 mins
N=9
n=
180
***
**
*
WT
hic-1
egl-21
egl-21; hic-1
E
PACh::NLP-21
Fluorescence Intensity (A.U.)
8000
4000
0
F
400
200

Figure S5. Mutants in *hic-1* do not affect Wnt transcriptional levels and are not involved in neuropeptide release. Related to Figure 3.

A. Representative images of coelomocytes of animals expressing mCherry tagged Wnt/CWN-2 and Wnt/LIN-44 in cholinergic neuron. The genotypes of the imaged animals are indicated in each panel.

B. The top panel shows a schematic representation indicating *C.elegans* expressing the P*unc-122*::GFP marker and Wnt::mCherry in the coelomocyte. The lower part of the figure indicates representative images of coelomocytes expressing P*unc-122*::GFP and CWN-2::mCherry or P*unc-122*::GFP and LIN-44::mCherry in different genotypes as indicated in each panel (The images are representatives of 10 images that were imaged per genotype).

C. Quantitative real time PCR graph for Wnt ligands *cwn-2*, *lin-44*, and the neuropeptide *nlp-21* in the genotypes; WT, *hic-1*, and *mig-14*. The experiment was performed thrice from independent RNA samples and the data was analyzed using two-way ANOVA.

D. Bar graph indicating the paralysis of *C. elegans* on Aldicarb at the 60 m time point. The genotypes assayed were; WT, *hic-1*, *egl-21* and *egl-21; hic-1*.

E. Representative images and quantitation of fluorescence intensity along the DNC in animals expressing fluorescently tagged NLP-21 in cholinergic motor neurons. WT (n=18) and *hic-1* (n=16) animals were analyzed. The data are shown as mean ± SEM and the p value calculated using two-tailed unpaired student's t-test.

F. Representative images and quantitation of fluorescence intensity in the coelomocytes of WT and *hic-1* mutant animals, expressing fluorescently tagged NLP-21 in cholinergic motor neurons. WT (n=55) and *hic-1* (58) were imaged. The data are shown as mean ± SEM and the p values calculated using two-tailed unpaired student's t-test. "*" $p<0.05$, "**" $p<0.01$, "***" $p<0.001$ and "ns" is not significant in all figures.

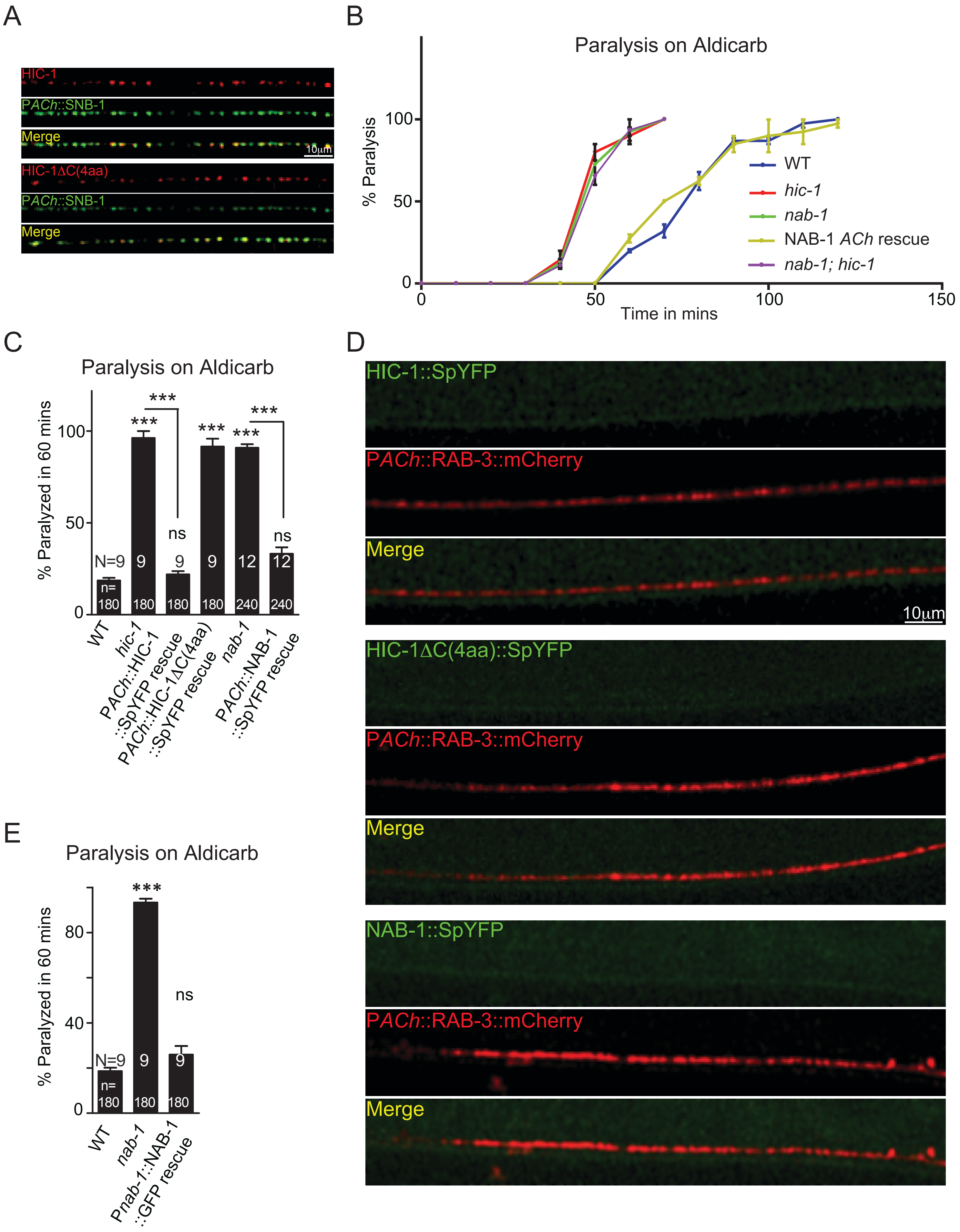

A
HIC-1
PACh::SNB-1
Merge
10µm
HIC-1ΔC(4aa)
PACh::SNB-1
Merge
B
Paralysis on Aldicarb
% Paralysis
Time in mins
WT
hic-1
nab-1
NAB-1 ACh rescue
nab-1; hic-1
C
Paralysis on Aldicarb
% Paralyzed in 60 mins
***
ns
N=9
n=
180
240
WT
hic-1
PACh::HIC-1::SpYFP rescue
PACh::HIC-1ΔC(4aa)::SpYFP rescue
nab-1
PACh::NAB-1::SpYFP rescue
D
HIC-1::SpYFP
PACh::RAB-3::mCherry
Merge
10µm
HIC-1ΔC(4aa)::SpYFP
PACh::RAB-3::mCherry
Merge
NAB-1::SpYFP
PACh::RAB-3::mCherry
Merge
E
Paralysis on Aldicarb
% Paralyzed in 60 mins
***
ns
N=9
n=
180
WT
nab-1
Pnab-1::NAB-1::GFP rescue

Figure S6. Analysis of the controls for the HIC-1 and NAB-1 interaction experiments. Related to Figure 5.

A. Expression of HIC-1 and HIC-1ΔC(4aa) in cholinergic synapses that are labeled with SNB-1. The number of animals imaged of each genotype are HIC-1 (n=12) and HIC-1ΔC(4aa) (n=15).

B. Line graph indicating the time course of paralysis on Aldicarb of *C. elegans* of the following genotypes: WT, *hic-1*, *nab-1*, *nab-1*; P*ACh*::NAB-1 and *hic-1; nab-1*. Each data point is represented as mean ± SEM.

C. Bar graph illustrating the paralysis on Aldicarb at the 60 m time point for the BiFC constructs used in Figure 5. The genotypes analyzed were WT, *hic-1*, *hic-1*; P*ACh*::HIC-1::SpYFP, *hic-1*; P*ACh*::HIC-1ΔC(4aa)::SpYFP, *nab-1* and *nab-1*; P*ACh*::NAB-1::SpYFP.

D. Representative images of the DNC in transgenics expressing RAB-3::mCherry along with each of the following constructs; HIC-1::SpYFP, HIC-1ΔC(4aa)::SpYFP and NAB-1::SpYFP, all of which were individually expressed in cholinergic neurons.

E. Bar graph illustrating paralysis on Aldicarb at the 60 m time point for the following strains; WT, *nab-1* and nab-1; P*nab-1*::NAB-1::GFP.

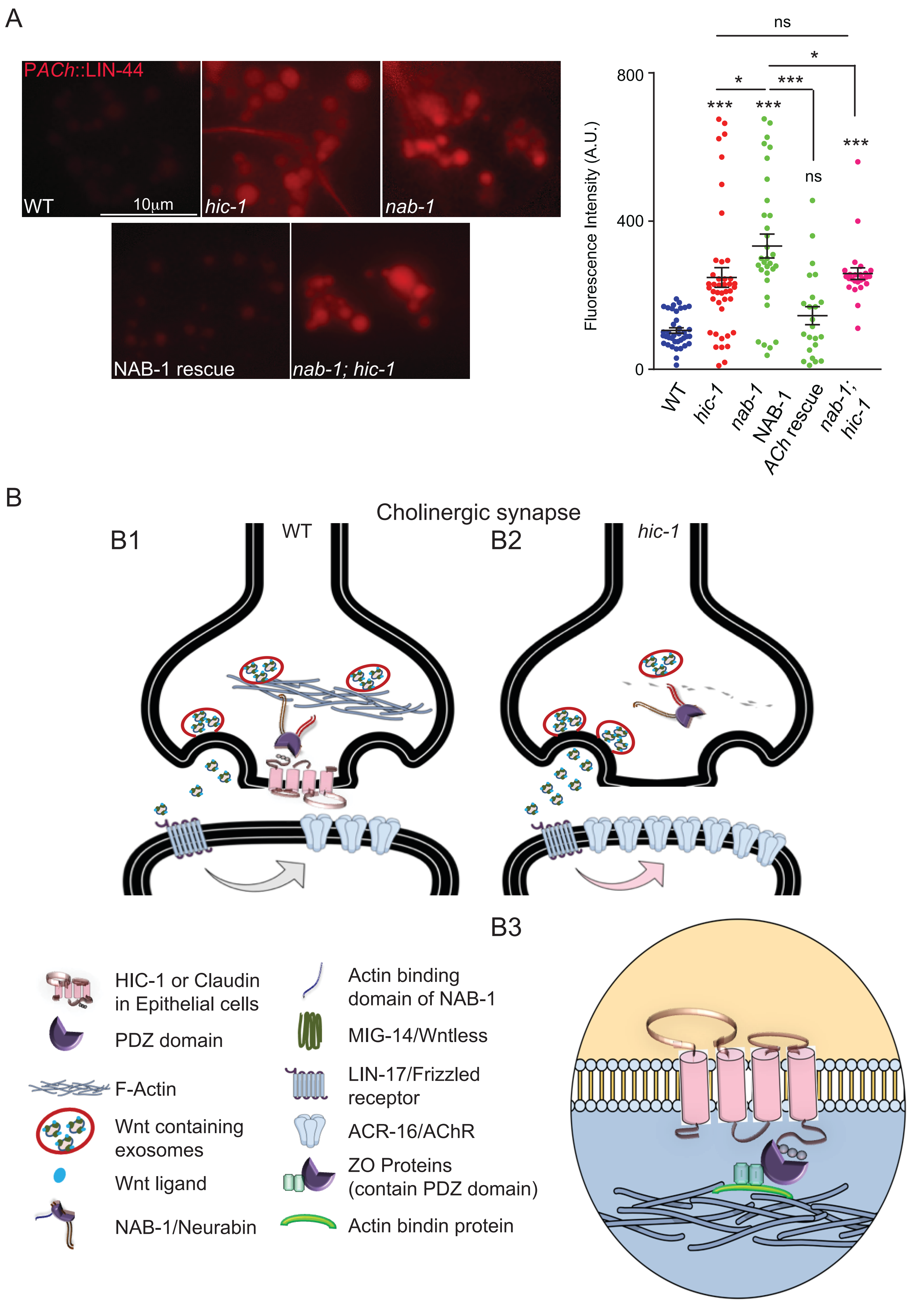
A
PACh::LIN-44
WT
10μm
hic-1
nab-1
NAB-1 rescue
nab-1; hic-1
Fluorescence Intensity (A.U.)
800
400
0
ns
*
*
***
***
***
***
ns
***
WT
hic-1
nab-1
NAB-1 ACh rescue
nab-1; hic-1
B
Cholinergic synapse
B1
WT
B2
hic-1
B3
HIC-1 or Claudin in Epithelial cells
PDZ domain
F-Actin
Wnt containing exosomes
Wnt ligand
NAB-1/Neurabin
Actin binding domain of NAB-1
MIG-14/Wntless
LIN-17/Frizzled receptor
ACR-16/AChR
ZO Proteins (contain PDZ domain)
Actin bindin protein

Figure S7. Mutants in *hic-1* and *nab-1* show increased LIN-44 secretion and possible model. Related to Figure 7 and Discussion.

A. Representative images and quantitation of fluorescence intensity in coelomocytes of animals expressing Wnt/LIN-44 tagged to mCherry in cholinergic neurons. The genotypes imaged were; WT (n=38), *hic-1* (n=42), *nab-1* (n=32), *nab-1*; P*ACh*::NAB-1 (n=23) and *nab-1; hic-1* (n=25). The Data are represented as mean ± SEM and p values calculated using one-way ANOVA and Bonferroni's Multiple Comparison Test.

B. Illustrations in B1 and B2 depict the possible function of HIC-1 at the NMJ of *C.elegans*. This model is based on previously published results (Jensen et al., 2012; Pandey et al., 2017) and the results presented in this manuscript. Please note that in this model we have focused on the role of HIC-1 in Wnt release.

B1. Illustrates the WT scenario where HIC-1 is expressed in cholinergic neurons and allows for normal Wnt release and hence normal AChR/ACR-16 levels at the NMJ.

B2. Depicts *hic-1* mutants that show increased Wnt release from cholinergic neurons and a causative increase in AChR/ACR-16 levels at the NMJ.

B3. Indicates the similarity between HIC-1 function at the NMJ and claudin function in epithelial cells. The intracellular region of HIC-1 appears to functions like a claudin by affecting the actin cytoskeleton through a PDZ domain molecule NAB-1, similar to the intracellular functioning of claudins in epithelial cells (reviewed in (Gunzel and Yu, 2013)).

Table S1. Primers and constructs. Related to Experimental procedures.

**Plasmid**	**Oligos**	**Description**
**pBAB# 0101:** P*hic-1*::mCherry	VT156 (*PstI*) AAAACTGCAGcctgcggagatatggagtctcaaaga caagg<br>VT86 (*BamHI*) CGCGGATCCcatggtttggtggagtttgg	2.4 Kb upstream of the *hic-1* gene was amplified from genomicDNA and cloned into pPD49.26 vector containing mCherry.
**pBAB# 0102:** P*hic-1*::HIC-1::mCherry	VT111 (*PstI*) AACTGCAGggcgttttaggctttcagaa<br>VT112 (*BamHI*) CGCGGATCCcattgcaggtatgacaagaagac	A 3.1kb of the whole genomic region of *hic-1* (Promoter and gene) were amplified and cloned upstream to mCherry in the pPD49.26 vector.
**pBAB# 0103:** P*rab-3*::HIC-1	VT48 (*XhoI*) CCGCTCGAGcggatgcatcatcatcaccatcacgcacc<br>VT49 *(BglII)* GAAGATCTTCttatcggtattccgagtatgctccatatccagtt<br>VT149 (*AgeI)* CTCTCTACCGGTcttcagatgggagcagtggac<br>VT150 (*SbfI)* CTCTCTCCTGCAGGgcctgctttttgtacaaacttgtcatct	The genomic region of *hic-1* was cloned in the pCFJ910 vector under *XhoI* and *BglII* sites. The promoter of *rab-3* was cloned upstream to HIC-1 using *AgeI* and S*bfI* sites.
**pBAB# 0104:** P*let-413*::HIC-1	VT119 (*AflII*) AGACTTAAGccttggtagcagcacagcag<br>VT120 (*XhoI*) CCGCTCGAGcggccatcttctgcttcttcttctcc	The promoter of *let-413* was amplified from *C. elegans* genomic DNA and cloned in the PCFJ910 vector using *AflII* and *XhoI* restriction sites to drive the expression of HIC-1 in the epithelial tissue.
**pBAB# 0105:** P*myo-3*::HIC-1	DM11 (*BamHI*) CGCGGATCCatgcatcatcatcaccatcacgc<br>DM12 *(NheI)* CTAGCTAGCgacgaattcaactgagcttgtacaattgtc<br>DM13 (*XbaI*) CTAGTCTAGAggctgcaacaaagatcagg<br>DM14 (*BamHI*) CGCGGATCCggagaacaatggtaaagcgtgg	The cDNA and the 3'UTR of HIC-1 was cloned in pPD49.26 vector in *BamHI* and *NheI* restriction sites. The *myo-3* promoter was amplified from the PCF104 vector and cloned upstream to HIC-1.
**pBAB# 0106:** Punc-*17*::HIC-1	DM09 (*SbfI*) CTCTCTCCTGCAGGgtcgaccatgacaaagtggtgacac<br>DM10 (*BamHI*) CGCGGATCCgactccaccgagttaccttaaaaaattgc	The *unc-17* promoter was cloned upstream to HIC-1 in pPD49.26 vector using *SbfI* and *BamHI* restriction sites to drive expression in the cholinergic neurons.
**pBAB# 0107:** P*unc-25*::HIC-1	VT245 (*SbfI*) CCTGCAGGccgacttaagggtcaaaagccg<br>VT246 (*BamHI*) CGCGGATCCagcaactacacaactgagccacc	P*unc-17* was replaced with the *unc-25* promoter in pBAB#106 plasmid by using the same restriction sites.
**pBAB# 0108:** P*unc-17*::HIC-1ΔC(4aa)	DM11 (*BamHI*) CGCGGATCCatgcatcatcatcaccatcacgc<br>VT182 (*NheI*) CTAGCTAGCttagtatgctccatatccagttgcacttcc	The cDNA of HIC-1 was amplified without the last 12 nucleotide bases encoding four amino acids (Putative PDZ binding motif) and cloned into pBAB# 0106 by replacing the full length HIC-1 gene.
**pBAB# 0109:** P*unc-17*::CWN-	VT 239 (*SphI*) CATGCATGCgtcgaccatgacaaagtggtgacac<br>VT 240 (*AvrII*) CTCTCTCCTAGGgactccaccgagttaccttaaaaa	The cDNA of the CWN-2 gene was cloned under *AvrII* and

2::mCherry	attgc<br>VT 241(*AvrII)* CTCTCTCCTAGGatgattccacggagaagttgttggc<br>VT242 (*BamHI*)<br>CGCGGATCCtttacaaatatccacttgtgtcacattattgcaagtttgacataccac	*BamHI* sites in the pPD49.26 vector containing mCherry. The *unc-17* promoter was cloned upstream to it using *SphI* and *AvrII* sites to drive the expression of CWN-2 in cholinergic motor neurons.
**pBAB# 0110:** P*unc-17*::NAB-1::Linker::VN173	VT199 (*SbfI*) NAB-1 Forward<br>cctgcaggatgacaacggcttccgagctgc<br>VT188 Linker-NAB-1 Reverse<br>atggttcatcactttctgtttcagatcgttcggaattttgcacgccgggcgcatgggaattgt gtgtgcaatgactcggg<br>VT189Linker Forward VN173<br>cgcccggcgtgcaaaattccgaacgatctgaaacagaaagtgatgaaccatgtgagca agggcgaggagc<br>VT190 (*KpnI*) VN173 Reverse<br>ggggtacctcactcgatgttgtggcggatcttgaagttggc	Full sequence and description are given in table S2.
**pBAB# 0111:** P*unc-17*::HIC-1::Linker::VC155	VT183 *(BamHI*) HIC-1 Forward<br>cgcggatccatgcatcatcatcaccatcacgcacc<br>VT184 HIC-1 Linker Reverse<br>atggttcatcactttctgtttcagatcgttcggaattttgcacgccgggcgtcggtattccga gtatgctccatatcc<br>VT185 Linker VC155 Forward<br>cgcccggcgtgcaaaattccgaacgatctgaaacagaaagtgatgaaccatcagaaga acggcatcaaggcc<br>VT186 (*NheI*) VC155 Reverse<br>ctagctagcttacttgtacagctcgtccatgccg	Full sequence and description are given in table S2.
**pBAB# 0112:** *Punc-17*:: HIC-1ΔC(4aa)::Linker:VC155	VT183 (*BamHI)* HIC-1 Forward<br>cgcggatccatgcatcatcatcaccatcacgcacc<br>VT200 Linker HIC-1ΔC(4aa) Reverse<br>atggttcatcactttctgtttcagatcgttcggaattttgcacgccgggcggtatgctccata tccagttgc	The cDNA of HIC-1 was replaced with HIC-1ΔC(4aa) using the same restriction sites used for cloning HIC-1 in pBAB# 0111
**pBAB# 0113:** P*unc-17*:: GFP-UtrCH	VT233 (*BamHI*) cgcggatccatggtgagcaagggcgaggag<br>VT234 (*NheI*) ctagctagcttagtctatggtgacttgctgagg	The GFP-UtrCH sequence was amplified from plasmid #26740 (Addgene) and cloned into pBAB# 0105 by replacing HIC-1 using *BamHI* and *NheI* sites.
**pBAB# 0114:** P*unc-25*:: GFP-UtrCH	VT245 (*SbfI*) cctgcaggccgacttaagggtcaaaagccg<br>VT246 (*BamHI*) cgcggatccagcaactacacaactgagccacc	Promoter of *unc-25* was replaced with *unc-17* promoter in pBAB# 0113 using *SbfI* and *BamHI* sites.
**pBAB# 0115:** P*hic-1*::HIC-1ΔC(4aa):: mCherry	VT255 (*PstI*) AAAACTGCAGcctgcggagatatggagtctcaaagacaagg<br>VT256 (*BamHI*) CGCGGATCCttagtatgctccatatccagttgcacttcc	The HIC-1 gene was replaced with HIC-1ΔC(4aa) in pBAB# 0102 using *PstI* and *BamHI* sites.
**Genotyping Primers**		
hic-1(ok3475)	External forward agcaggtaacaagt<br>Internal forward gagtatgctccatatccagtt<br>Common reverse accacaaactactctgaact	PS88<br>PS89<br>PS90
nab-1(ok943)	External forward atcgcaattttctccattcg	VT204

	External reverse<br>Internal forward<br>Internal reverse	gcgtacttctttcggagtgg<br>tattccgattccacacagca<br>cgatgcgtcaatttatgtgg	VT205<br>VT206<br>VT207
acr-16(ok789)	External forward<br>Internal forward<br>Common reverse	ggtagccctccaacaaataattacg<br>gggaaagtttacagtgacaactgtgg<br>gcatatgtcaagttgtgaccggg	VT158<br>VT159<br>VT160

Table S2. Sequences of the BiFC experimental constructs. Related to Figure 6.

**pBAB# 0110:**<br><br>ATGACAACGGCTTCCGAGCTGCTGTCAGACGACGCAAGAGCTCGATTCT<br>CACATACAAAGGCTCTTTTTGAGCAGCTTGAGAGACAGCAAGACGTTCC<br>ATCATTCTACTCTCCACGTCTTCAACGTCAACCACCACCTCCATTACCCC<br>CAAAACCTCCATCTCAATGTCCGCCGAGTCCGATGTCACAGGTCGAACG<br>AAATTTCTCTGATTTGGCCGCGGATCTCGATCGGATCCAATCATCGCCGG<br>CAACGTCTAGGTTTATACAAAATAAATCATCAACTCTTCCGTCATCATAT<br>TCCGATTCCACACAGCAGTATTCGTTCGAAAATACGGTTGCCACGTCGCA<br>ATATGGAGGACTTCAAACCAACAACAATAATAATAATATTAATATGAATT<br>CATTTGAACCCTATTGGAGAAATGGATCAATATATCGGAGGCAATTCGAA<br>GGAAATGGCAAACCTTTTGGCGAGGAAAACGATGGAATCTCACCAACGAC<br>GAATGGAGTGAAGCACACGACATACGCCGTCGTCAAAACACCAAAGGAAA<br>CAGAACTTGAAACAACATCAGAAAACAGAGGACTCATGGAAACACGGAGG<br>GGTCTGAGTCCAGAGAGGCCTGACGTGGATTTAACGAATCGAAAGGTCTCGT<br>TCAGCACTGCACCTATTCCGGTCTTCTCGGCGTTTTCCGTTGAAGACTACGAT<br>CGCAAGAATGAAGACATTGATCCGGTGGCAAGTTGTGCTGAATATGAATTGG<br>AGAGACGACTTGAAAGAATGGATTTGTTTGAAGTTGATTTGGAAAAGGGTGC<br>CGAGGGACTTGGAGTCTCTATTATTGGTATGGGTGTTGGTGCAGACTCGGGTC<br>TTGAAAAGCTGGGAATCTTTGTAAAGAGCATCACACCTGGCGGAGCTGTTCAT<br>CGAGATGGACGAATTCGTGTCTGCGATCAAATTGTTTCAGTCGATGGAAAGAG<br>TTTGGTCGGTGTCTCACAGTTGTATGCTGCAAACACGTTGAGATCCACCAGCA<br>ATCGAGTCACTTTCACAATTGGTCGCGAACAAAACCTTGAAGAATCCGAAGT<br>TGCTCAGCTAATTCAACAAAGTCTTGAACACGACAGAATGAGAATGATGGGA<br>GACGAGGAAGACGATATCGAAGAGCCGCCACCACCACCATCTCAAATGCCAC<br>AGCTACCAATTGAACAGGATCGTCCGACAACTTCAATGATCACAAAAGAAGA<br>AATTGAGATTCGTTCGAAGATTGCTGCTCTTGAACTGGAACTTGATGTTACTC<br>ATAAGAAAGCAGAGCAATATCATGAAGTGTTGAGCTCAACGAAATCACATTG<br>TGATCAACTGGAAAAGCAGAATGAGCAGGCAAATTATATGATTAAAAACTAT<br>CAGGAAAGAGAAAAAGAGTTGTTGAATCGAGAAGAGAATCATGTGGAACAA<br>TTGAGAGATAAGGATGTACATTATGCATCATTAGTTCGTCAATTGAAAGAAC<br>GAATTGATGAGTTGGAATCGAAATTGGAAGAAGCTGAAGAGAGAAGGCATT<br>CTATTCAGAATCTTGAGCTTATCGAGTTGAGAGAGAAGTTAAAGGAGAAAGT<br>TGAGAAGAGGAATGAAGGAATGGCGTATAGACCAGGTGGAGAGCTTCCTCA<br>CGAGGATAAGGCTGTTATGGTTAATTTGGAGACTATTACTCCATCAACCAAA<br>AAAGATGCGGAAGTATCAGTTGGAAGTAGTTGGACAGAAGAGTACTCATCA<br>CCATGCGAGTCGCCAGTCCCTCGTATCTCCGAGCCAGCATCTCCGGCTCTTC<br>CACATAAATTGACGCATCGCAAACTTCTGTTCCCACTCCGAAAGAAGTACG<br>CCGAAAACGAGTTCTGGCGTGCCACGTGTCAACCAGTTGGTCTTCAAGCTCT<br>TCATTGGACGGTGGACGATGTTTGTCAGTTGCTCGTTTCAATGGGCTTGGAC<br>AAATATGTGCCAGAGTTCACCATTAATAAGATCGATGGAGCCAAGTTCCTC<br>GAGCTCGACGGAAATAAACTGAAGGCAATGGGAATTCAAAATCACTCGGA<br>TCGTTCTCTAATTAAAAAGAAAGTAAAAGGAATGAAGAATAAAATTGAAA<br>GAGAACGAAAACAGTTGGAACGAGAAAGCCGTACCCGAGTCATTGCACAC<br>ACAATTCCCATGcgcccggcgtgcaaaattccgaacgatctgaaacagaaagtgatgaaccatGTGAGCA<br>AGGGCGAGGAGCTGTTCACCGGGGTGGTGCCCATCCTGGTCGAGCTGGACG<br>GCGACGTAAACGGCCACAAGTTCAGCGTGTCCGGCGAGGGCGAGGGCGAT	The sequence shown here is for a construct where the Neurabin gene (black) and VN173 fragment of YFP gene (green) are separated by a linker sequence (red). The linker sequence was incorporated using primers VT188 and VT189.

GCCACCTACGGCAAGCTGACCCTGAAGCTGATCTGCACCACCGGCAAGCT<br>GCCCGTGCCCTGGCCCACCCTCGTGACCACCCTGGGCTACGGCCTGCAGTG<br>CTTCGCCCGCTACCCCGACCACATGAAGCAGCACGACTTCTTCAAGTCCGC<br>CATGCCCGAAGGCTACGTCCAGGAGCGCACCATCTTCTTCAAGGACGACGG<br>CAACTACAAGACCCGCGCCGAGGTGAAGTTCGAGGGCGACACCCTGGTGA<br>ACCGCATCGAGCTGAAGGGCATCGACTTCAAGGAGGACGGCAACATCCT<br>GGGGCACAGCTGGAGTACAACTACAACAGCCACAACGTCTATATCACCG<br>CCGACAAGCAGAAGAACGGCATCAAGGCCAACTTCAAGATCCGCCACA<br>ACATCGAGTGA	
**pBAB# 0111:**<br>ATGCATCATCATCACCATCACGCACCATTCGCCATTGCTGGCCTTCTGA<br>TTATTGTTACAATACTAACCGGAGTGGGAACTTTTACAAATTATTGGGG<br>TGTTTCTGGAAACTTGCACATGGGAATTTATCAATGGGGACAGGCAGG<br>CGCGAACAGAAGTTTCCAAAGAAGTGCGGGGTGGCTGCAATGTGTCGT<br>GGTTTGTCAATTAATGGCATTCTCATTTGAGCTCTTGTTTTGTCTTCTTG<br>TCATACCTGCAATAGTTTTCCGAAGAATGATGCCGGTTCATGCAGCGT<br>GTACTCTTCTCTCCTTGATCATATTCATTTTACTTCTTATTTCCATCATT<br>GTATTCGCGGCAAATATTGGAAGCTTCTATTACAACGCATTGATTTCG<br>TTAAAGCTCGGATGGTCATGGGGAATCACTTTGGCTGCTACAATTCTT<br>TCATTTCTTTTACTACTAGTATCTGGAAGTGCAACTGGATATGGAGCA<br>TACTCGGAATACCGAcgcccggcgtgcaaaattccgaacgatctgaaacagaaagtgatgaaccat<br>CAGAAGAACGGCATCAAGGCCAACTTCAAGATCCGCCACAACATCGA<br>GGACGGCGGCGTGCAGCTCGCCGACCACTACCAGCAGAACACCCCCA<br>TCGGCGACGGCCCCGTGCTGCTGCCCGACAACCACTACCTGAGCTAC<br>CAGTCCAAACTGAGCAAAGACCCAACGAGAAGCGCGATCACATGGT<br>CCTGCTGGAGTTCGTGACCGCCGCCGGGATCACTCTCGGCATGGACG<br>AG CTGTACAAGTAA	The sequences shown here is for a construct where the HIC-1 gene (black) and VC155 fragment of YFP gene (green) is separated by a linker sequence (red). The linker sequence was added in the primers VT184 and VT185 as highlighted.
